# Investigating for Whom Brief Substance Use Interventions Are Most Effective: An Individual Participant Data Meta-analysis

**DOI:** 10.1007/s11121-023-01525-1

**Published:** 2023-05-03

**Authors:** Maria L. Schweer-Collins, Nicholas J. Parr, Richard Saitz, Emily E. Tanner-Smith

**Affiliations:** 1 Prevention Science Institute, University of Oregon, University of Oregon, 97403-6217 Eugene, OR USA; 2 U.S. Department of Veterans Affairs Evidence Synthesis Program Coordinating Center, VA Portland Health Care System, 3710 SW U.S. Veterans Hospital Rd, 97239 Portland, OR USA; 3 Department of Community Health Sciences, School of Public Health, Boston University, 801 Massachusetts Ave, 4th Floor, 02118 Boston, MA USA; 4 Clinical Addiction Research and Education Unit, Section of General Internal Medicine, Boston University School of Medicine, Boston, USA; 5 Grayken Center for Addiction, Boston Medical Center, Boston, USA; 6 HEDCO Institute for Evidence-Based Educational Practice, University of Oregon, University of Oregon, 1215, 97403-1215 Eugene, OR USA

**Keywords:** Brief intervention, Meta-analysis, Individual participant data, Primary care, Systematic review

## Abstract

**Abstract:**

Prior research suggests that brief interventions (BIs) for alcohol and other drug use may vary in effectiveness across patient sociodemographic factors. The objective of this individual participant data (IPD) meta-analysis was to explore for whom BIs delivered in general healthcare settings are more or less effective. We examined variability in BI effects by patient age, sex, employment, education, relationship status, and baseline severity of substance use using a two-stage IPD meta-analysis approach. All trials included in a parent aggregate data meta-analysis (*k* = 116) were invited to contribute IPD, and 29 trials provided patient-level data (12,074 participants). Among females, BIs led to significant reductions in binge alcohol consumption ($\overline{g }$ = 0.09, 95% CI [0.03, 0.14]), frequency of alcohol consumption ($\overline{g }$ = 0.10, 95% CI [0.03, 0.17]), and alcohol-related consequences ($\overline{g }$ = 0.16, 95% CI [0.08, 0.25]), as well as greater substance use treatment utilization ($\overline{g }$ = 0.25, 95% CI [0.21, 0.30]). BIs yielded larger reductions in frequency of alcohol consumption at 3-month follow-up for individuals with less than a high school level education ($\overline{g }$ = 0.16, 95% CI [0.09, 0.22]). Given evidence demonstrating modest BI effects on alcohol use and mixed or null findings for BI effects on other drug use, BI research should continue to investigate potential drivers of effect magnitude and variation.

**Protocol registration details:**

The protocol for this review was pre-registered in PROSPERO #CRD42018086832 and the analysis plan was pre-registered in OSF: osf.io/m48g6.

**Supplementary Information:**

The online version contains supplementary material available at 10.1007/s11121-023-01525-1.

## Introduction

Substance use disorders and related injuries are among the top five causes of death for adolescents in North America (World Health Organization, 2020) and among the top 20 causes of premature mortality for North American adults (Pan American Health Organization [PAHO], 2021). In 2019 alone, there were 85,984 deaths in North America attributed to substance use, accounting for almost 50% of substance use disorder deaths globally (PAHO 2021). The impact of substance misuse necessitates timely, effective substance use prevention and treatment. Because general healthcare settings (e.g., emergency department or primary care clinics) are more highly utilized than specialist care settings, they may allow for the broadest reach of prevention services aimed at reducing harmful substance use. Moreover, because individuals with a variety of healthcare needs (potentially unrelated to substance misuse) commonly present in general healthcare settings, there may be increased opportunity in these settings for healthcare professionals to screen for and discuss alcohol and other drug use with patients with previously undetected substance misuse.

Brief interventions (BIs) are one evidence-based and widely implemented strategy for preventing substance use problems (Solberg et al., 2008; Tanner-Smith & Grant, 2019). Here, BIs are defined as time-limited and structured interventions that aim to produce positive change in substance use behaviors or their determinants. BIs are commonly delivered within a screening, brief intervention, and referral to treatment (SBIRT) model to non-treatment-seeking individuals who may report harmful levels of alcohol use (SAMHSA, 2013). In this population, a key goal of the BI is to assist patients to develop awareness of substance use levels that are problematic and to enhance motivation to reduce substance use (SAMHSA, 2013; Tanner-Smith & Grant, 2019). BIs can be incorporated into a variety of settings given their brevity, low-cost, flexible structure, and their ability to be delivered by professionals and non-clinical interventionists alike (Kunz et al., 2004; Neighbors et al., 2010; Wutzke et al., 2001). Together these attributes make BIs an attractive prevention approach for substance use problems.

A large body of literature has examined the effectiveness of BIs for alcohol and other drug use problems. Generally consistent, modest effects have been found for BIs targeting alcohol use (Bertholet, 2005; Kaner et al., 2018; Tanner-Smith & Lipsey, 2015; Tanner-Smith et al., 2021). Less research has examined the effectiveness of BIs delivered in primary care settings for drugs other than alcohol, and here BIs often show null or inconsistent effects (Saitz, et al., 2014; Saitz, 2020; Tanner-Smith et al., 2021; Young et al., 2014). Because findings of BI trials have been inconsistent, especially for drugs other than alcohol, there have been calls to more carefully examine variability in BI effectiveness and to consider alternate ways to address unhealthy substance use (Hingson & Compton, 2014; Saitz, 2020).

Several rigorous randomized controlled trials have examined variation in the effectiveness of BIs according to patient characteristics (e.g., sociodemographic and psychosocial contextual risk factors), suggesting that some patient subgroups are more or less likely to benefit from this form of low-intensity intervention. For example, in a sample largely composed of persons with low income, high unemployment, and high rates of mental and physical health comorbidities, there was no evidence of positive BI effects on drug use, heavy episodic drinking, and drug use consequences at 6 months post-treatment regardless of the length of BI (i.e., BI lasting 15 min vs. a BI that included a 35–40-min motivational interviewing enhancement; Saitz et al., 2014). In contrast, other rigorous evidence suggests that BIs delivered in primary care settings are effective at reducing risky drug use at 3 months post-intervention in racially and ethnically diverse adult populations (Gelberg et al., 2015, 2017). In another trial conducted with women experiencing high degrees of psychosocial risk (e.g., interpersonal violence, histories of childhood sexual abuse) and mental health comorbidities (e.g., depression and post-traumatic stress disorder), a BI delivered in an emergency department setting had no effect on alcohol use outcomes (Rhodes et al., 2015). Other evidence suggests that BIs may have little or no effect for patients experiencing psychiatric comorbidities (Walton et al., 2008). Prior research has also demonstrated that BI effects on drug use are greater in treatment-seeking patients (versus screened samples), and that BIs may be less effective for persons experiencing greater severity of substance use (Patnode et al., 2020; Saitz, 2010). Evidence from prior systematic reviews and meta-analyses also suggests variability in BI effects by patient characteristics. For instance, results from a recent overview of 24 systematic reviews of alcohol-focused BIs found little evidence of benefit among adolescent and aging adult populations, women, persons experiencing unstable housing, and minoritized racial and ethnic populations (O’Donnell et al., 2014).

Although these findings suggest variability in effectiveness of BIs according to participant characteristics, to our knowledge, all systematic reviews or meta-analyses on BIs delivered in general healthcare settings have analyzed aggregate, or study-level, data (AD). AD meta-analyses are best able to examine moderators that do not vary within studies (e.g., methodological features of the study or features of the intervention in each study). Analyzing moderators that vary within studies, such as participant age or other demographic characteristics, using AD (i.e., summary values like means or proportions) can misrepresent true moderation effects (Parr et al., 2019; Tanner-Smith et al., 2022). Moreover, because any given subgroup of patients within a trial may be quite small, individual trials are often underpowered to detect meaningful subgroup differences. The field is thus limited in its ability to determine which patients benefit most from BIs, hampering efforts to deliver substance use interventions to patients for whom they are most likely to be effective.

To address these limitations, research on BIs for alcohol and other drug use has begun to use individual participant data (IPD) meta-analysis in areas including BIs for unhealthy college drinking (Huh et al., 2015; Huh et al., 2022; Mun et al., 2022). IPD meta-analysis can be used to harmonize and analyze data on a large number of individual participants, and allow for questions about patient-level variation in intervention effects to be investigated. Other advantages of IPD meta-analysis compared with AD meta-analysis can include greater precision of effect size estimates, and improved consistency and rigor of treatment-covariate (i.e., moderation) models through direct re-analysis of primary study data (Cooper & Patall, 2009). These relative advantages can yield clearer insights into variability in intervention effects and provide more actionable findings to practitioners and policymakers. The current study is an effort to consolidate and synthesize IPD from multiple BI trials to examine which patient characteristics might moderate the effectiveness of alcohol- and drug-focused BIs delivered in general healthcare settings.

### Study Objective

The primary objective of this IPD meta-analysis was to explore for whom BIs delivered in general healthcare settings are more or less effective across a range of outcomes: alcohol and other drug use, tobacco use, alcohol and other drug-related consequences, mental and physical health, emergency department use, substance use treatment utilization, and readiness to change substance use behaviors. We examined variability in BI effects by patient age, sex, employment, education, relationship status, and baseline severity of substance use at three follow-up timepoints (3 months, 6 months, and 12 months post-baseline).

## Methods

### Protocol and Pre-registration

The protocol for this IPD meta-analysis was pre-registered in the PROSPERO registry #CRD42018086832 (Tanner-Smith et al., 2018) and the analysis plan was pre-registered on OSF (Tanner-Smith et al., 2020; osf.io/m48g6). Any deviations from the pre-registered protocol are justified and documented on OSF.

### Study Eligibility Criteria and Search Strategy

This IPD meta-analyses used data from a larger parent project that also involved an AD meta-analysis (Tanner-Smith et al., 2021). Study eligibility for the parent project included the following: a randomized controlled trial (RCT) that used a no treatment, straw-person, sham intervention, or practice as usual comparison condition to evaluate the effects of an alcohol or other drug-focused BI delivered in a general healthcare setting; the study was reported in 1990 or thereafter; the study reported on at least one post-BI outcome of substance use or substance-related consequences; the BI in each study must have been delivered in four or fewer sessions to participants recruited in a general healthcare setting (e.g., primary care, general hospital, emergency department).

To identify trials meeting the inclusion criteria, we completed the following: (a) searched the following databases through March 31, 2020: PubMed; Nursing/Academic Edition (EBSCO host); ERIC, Applied Social Sciences Index and Abstracts, Dissertations & Theses Global, Social Services Abstract (ProQuest host); PsycINFO (PsycNET host); Cochrane Central Register of Controlled Trials; the WHO International Clinical Trials Registry; and the NIH RePORTER website (see Supplemental Material 1 for the full PubMed search strategy); (b) reviewed the bibliographies of all screened and eligible studies as well as all prior reviews and meta-analyses for additional studies; and (c) performed hand-searches of the 1990–2020 tables of contents in the journals *Addiction*, *Addictive Behaviors*, *Campbell Systematic Reviews*, and *Journal of Studies on Alcohol and Drugs*. See Supplemental Material 1 for the search terms used.

### Study Selection

To identify eligible studies and extract aggregate level study data, a team of research assistants (RAs) were trained and supervised by senior author ETS. First, reviewers screened titles and abstracts to remove ineligible studies. Any study that was determined to be potentially eligible by at least one reviewer in this stage proceeded to the second state of screening. During the second stage of screening, two reviewers independently reviewed the full text to determine study eligibility. Finally, at the third stage, two reviewers independently extracted AD for eligible trials. When disagreements in the second and third stages of screening and data extraction arose, they were resolved by senior author ETS. Data extraction was conducted using a standardized coding protocol and RAs entered data into a FileMaker Pro database. Aggregate study data extracted and coded in this database were primarily used to describe primary study characteristics and assess risk of bias.

### IPD Data Collection and Data Synthesis

Primary study investigators of all eligible trials included in the AD meta-analysis (Tanner-Smith et al., 2021) were contacted through email with an overview of the proposed IPD meta-analysis and an invitation to collaborate. Following initial contact, primary study investigators were sent a scripted email and offered their preference of either (1) a teleconference with the IPD meta-analysis principal investigator and project managers and/or (2) an email containing detailed information on the proposed IPD meta-analysis, a templated data sharing/data use agreement, information concerning the de-identification of data and protection of human subjects, and options for the secure transfer of data. In the case of no response, a series of reminder emails were sent every 2 weeks for 2 months. If no response was received, the IPD were recorded as unavailable due to investigator non-response. When available, IPD from primary studies were acquired directly from open-access data repositories.

All data cleaning and analysis were performed in the R environment (R Core Team, 2019; RStudio Team, 2019). Individual trial data were checked for consistency with published reports, including checking for missing values, comparing study sample size against published study reports and trial registries, and data distributions were checked for any missing, invalid, out of range, or inconsistent values. Baseline equivalence between primary study arms were checked using all available demographics. Data inconsistencies or missing data were discussed with relevant study investigators and corrected when necessary.^1^ Data were then recoded for consistency across all studies in terms of the directionality of outcomes and the levels of each moderator of interest (see Supplemental Material 2 for notes on data harmonization and coding of all moderators and outcome domains).

### Effect Sizes

Pretest-adjusted standardized mean differences for patient subgroups of interest (e.g., the marginal subgroup effect estimated from a model that included the interaction between the intervention condition and moderator of interest) were the effect sizes estimated for each individual trial during the first stage of analysis (the estimation of overall intervention effectiveness is reported in the project’s corresponding AD meta-analysis (Tanner-Smith et al., 2021)). In the second stage of analysis, these effect sizes were then synthesized into pooled pretest-adjusted standardized effects. Primary domains included alcohol consumption (frequency and quantity), binge alcohol use, other drug consumption (frequency and quantity), cannabis consumption, tobacco consumption, alcohol-related consequence, and other drug consequences. Secondary domains included physical health, mental health, substance use treatment utilization, emergency department utilization, and readiness to change.

### Moderator Domains

Data were harmonized and coded for the following moderator variables when available in primary studies.^2^ Age was harmonized into a three-level factor: adolescent (under 18); young adult (18–25); adult (26 or older). Housing status was a dichotomous variable with 0 representing being currently unhoused and 1 representing having stable housing. Employment status was a dichotomous variable with 0 representing being unemployed and 1 representing being currently employed half-time or greater. Education was a dichotomous variable with 0 representing have below a high school level of education and 1 representing having obtained a high school diploma/equivalent or greater. Note that adolescents below 18 years of age were not coded for education due to confounding with age, that is, that it would generally not have been possible for this subgroup to have obtained a high school education. Relationship status was coded as 0 representing a single relationship status, including being divorced, separated, or widowed; 1 was coded as being married, which could include marriage, a stable partnership, or having a current partner. Sex was coded dichotomously with 0 representing male and 1 representing female. Last, baseline severity of substance use was coded as a three-factor categorical variable, using the AUDIT or ASSIST. Low baseline severity of use was represented by an AUDIT score of 1–7 or ASSIST total score of 1–3; moderate baseline severity of use was represented by an AUDIT score of 8–15 or ASSIST total score of 4–26; last, high baseline severity of use was represented by an AUDIT score of 16 or above or an ASSIST total score of 27 or above.

### Study Reporting Quality, Risk of Bias, Design Assessment

**Fig. 1:**
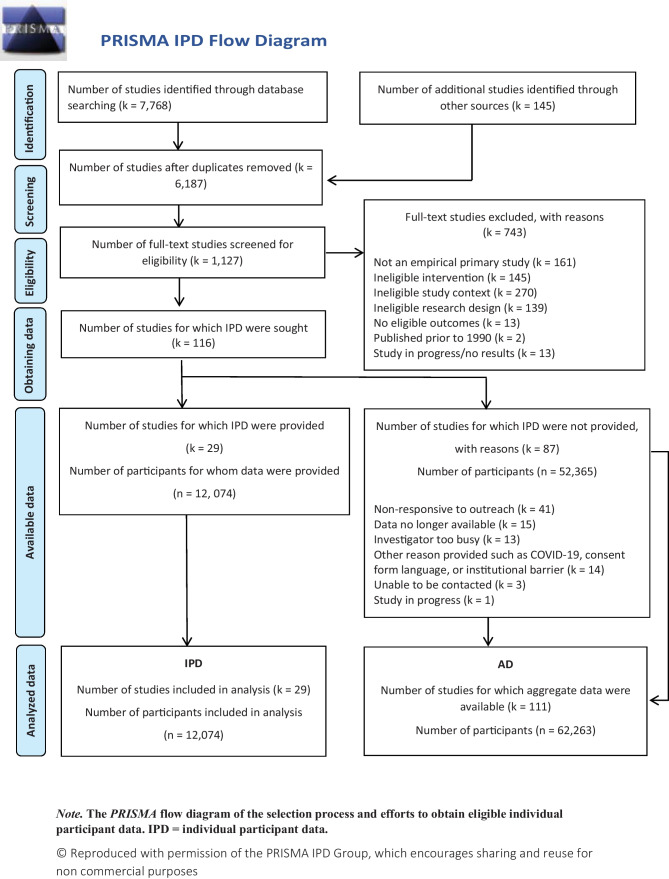
PRISMA IPD flow diagram. *Note.* The *PRISMA* flow diagram of the selection process and efforts to obtain eligible individual participant data. IPD, individual participant data. © Reproduced with permission of the PRISMA IPD Group, which encourages sharing and reuse for non-commercial purposes

For all included IPD studies, risk of bias assessments were collected using Cochrane’s risk of bias tool for RCTs (Higgins & Altman, 2008). Studies were given ratings of high, low, or unclear risk of bias in six domains, defined as low (all domains rated low), high (any domain rated high), and unclear (any domain rated unclear and no domains rated high). Publication bias was quantitatively and graphically assessed in the corresponding AD meta-analysis (Tanner-Smith et al., 2021). In the online supplement, we present descriptive comparisons between AD studies and studies that contributed IPD. In Fig. 1, we report information on the proportion of primary studies and primary study participants obtained, and the reasons primary study data were not available. The reporting of this IPD meta-analysis was guided by the standards of the Preferred Reporting Items for Systematic Reviews and Meta-Analyses of IPD (PRISMA IPD) Statement (Stewart et al., 2015).

### Analytic Strategy

Analysis of IPD was carried out using a two-stage modeling approach (Burke et al., 2017; Stewart et al., 2012; see Supplemental Material 3 for the pre-registered analysis plan). The two-stage approach was selected for interpretability and because it simplified handling of missing data at the study level (Koopman et al., 2007). The two-stage approach can be used to synthesize subgroup marginal means, which allowed us to present BI treatment effects for the subgroups of interest (e.g., men and women, individuals with low, moderate, and high severity of baseline substance use) in a clinically useful way. Before the first stage of analysis, missing data were recovered using multiple imputation. Imputation was carried out at the individual study level using all available study data. Predictor and outcome variables in all studies were then standardized so that model output was in the form of standardized regression coefficients.

In the first stage of the IPD meta-analysis, pretest-adjusted intervention effects and intervention by covariate interaction effects were estimated at the primary study level, resulting in marginal subgroup effect estimates. Due to this two-stage analytic strategy, only one treatment-covariate interaction was included in each model (e.g., age × treatment). In the second stage, marginal subgroup effect estimates were pooled across studies using random-effects meta-analysis models with standard inverse-variance weighting. Mean effect sizes and 95% confidence intervals are presented for each analysis. A Benjamini–Hochberg procedure for multiple comparisons (Benjamini & Hochberg, 1995) was implemented to control false discovery rates within an outcome domain.

Pooled mean effect sizes were estimated separately for the primary and secondary outcome domains and within each follow-up time-period (i.e., 1–3 months, 6 months, 9–12 months). To maintain independence of the effect size estimates in each analysis, only one effect size per individual participant dataset for each domain (e.g., frequency alcohol consumption, other drug consumption) and at each timepoint (e.g., 3-month, 12-month) was included in each analysis. For studies that reported multiple outcome effect sizes within domains (for example, weekly and monthly frequency of alcohol use), we used decision rules to select the effect size to be included in the analysis. Specifically, preference was given to effect sizes that were (1) continuous measures versus dichotomous measures; (2) more general measures (e.g., general mental health versus depression or anxiety scores); (3) the effect size reported for the time frame that was most consistently available across all studies; (4) the effect size that had a corresponding baseline score; and (5) the effect size specific to drug or alcohol consequences (i.e., an emergency department visit related to substance use versus all hospitalizations). Regarding dependences for studies with multiple treatment contrasts (e.g., control versus screening and assessment; control versus BI), we selected the treatment contrast between the most minimally intensive control group and the most intensive intervention. A sensitivity analysis was used to assess whether the contrast between the selected control and intervention conditions (e.g., screening and assessment) substantively altered study findings (see Supplemental Material 4 for results from the sensitivity analysis; findings were robust to this modeling decision). Publication bias was assessed using Orwin’s fail safe N and funnel plots (see Supplemental Material 8).

## Results

### Study and Participant Characteristics

In the parent AD meta-analysis, 116 trials met eligibility criteria (Tanner-Smith et al., 2021). While all eligible trials were invited to contribute IPD, 29 clinical trials provided patient-level data (Fig. 1; see Supplemental Material 5 for references of included trials and Supplemental Material 6 for study characteristics of all included studies). In total, 12,074 participants were included in this meta-analysis; 6,577 (54.5%) received a BI for alcohol or other drug use and 5497 (45.5%) were allocated to a no treatment, straw-person, sham intervention, or practice as usual comparison condition, with the most common control being treatment as usual (45%). Trials were conducted in the USA and Canada (*k* = 17; 55%) and internationally (*k* = 13, 45%). The majority of trials were conducted with a sample at elevated risk for substance use behavior (97%); most trials used an individual, randomized controlled trial design (90%). Most trials targeted mixed alcohol and other drug use (*k* = 11, 38%) or alcohol only (*k* = 14; 48%), with only four trials targeting specific drugs (14%). Across the trials, BIs were most commonly delivered by behavioral specialists (*k* = 10, 34%), via in-person formats (*k* = 25, 86%), and at emergency department (*k* = 10, 34%) or community healthcare settings (*k* = 11, 38%). Trials commonly followed up with participants at 3 months post-treatment (72%), although some trials had longer follow-ups at 6 months (55%) or 12 months post-treatment (48%; see Table 1). Statistical comparisons between the IPD sample and the AD sample are also reported in Table 1 to assess for the representativeness of the IPD sample against the known body of studies reflected in the AD meta-analysis (see Supplemental 7 for statistical comparisons between the IPD sample and the AD sample that does not include the IPD studies).

**Table 1:** Descriptive comparison of characteristics of studies and interventions included in qualitative and quantitative aggregate data and individual participant data syntheses (AD *k* = 116; IPD *k* = 29)

Study and design characteristics	% (*n*) ^a^			Intervention features	% (*n*) ^a^		
	AD	IPD	*p* ^g^		AD	IPD	*p* ^g^
Country/region ^b^			> .05	Setting ^b^			> .05
Asia	4 (5)	3 (1)		Emergency department	41 (48)	34 (10)	
Australia/New Zealand	8 (9)	3 (1)		Community	34 (39)	39 (11)	
South Africa	4 (5)	3 (1)		University	22 (25)	21 (6)	
South America	3 (3)	7 (2)		Outpatient	11 (13)	14 (4)	
U.S./Canada	59 (68)	55 (16)		Inpatient	5 (6)	7 (2)	
* U.S. Midwest*	22 (15)	-		Private provider	9 (11)	7 (2)	
* U.S. Northeast*	43 (29)	28 (8)		Student health center	6 (7)	0 (0)	
* U.S. South*	10 (7)	7 (2)		Other	18 (21)	7 (2)	
* U.S. West*	21 (14)	19 (4)		Modality ^c^			> .05
* Multiple U.S. regions*	4 (3)	-		In-person	75 (88)	86 (25)	
Western Europe	21 (24)	28 (8)		Computer/tablet/smartphone	20 (24)	7 (2)	
Multiple	2 (2)	-		Telephone	5 (6)	7 (2)	
Sample type ^b^			> .05	Booster ^c^			
Screened/elevated risk	97 (112)	93 (27)		Booster delivered	33 (40)	24 (7)	> .05
Universal/unscreened	3 (4)	7 (2)		No. boosters; median (range) ^d^	1 (1–4)	1 (1–2)	
Design ^b^			> .05	Duration (minutes); *M* (*SD*) ^c^	26.9 (25.1)	21.0 (19.5)	
RCT	90 (104)	90 (26)		Components ^c, e^			> .05
Cluster RCT	10 (12)	10 (3)		Advice	62 (75)	59 (17)	
Comparison group type ^c^			> .05	Information booklet	59 (71)	55 (16)	
Treatment as usual/usual care	45 (55)	59 (17)		Decisional balance exercise	32 (39)	41 (12)	
General health booklet	34 (41)	24 (7)		Goal-setting exercise	54 (66)	66 (19)	
Sham intervention	7 (8)	10 (3)		Homework activity	4 (5)	0 (0)	
No pretest assessment usual care	3 (4)	-		Personalized normative feedback	75 (89)	72 (21)	
Other	11 (14)	7 (2)		Training	16 (19)	10 (3)	
Attrition; *M* (*SD*)				Referrals	26 (30)	28 (8)	
Overall ^b^	0.25 (0.18)	0.22 (0.12)		Video	4 (5)	7 (2)	
Differential ^c^	0.05 (0.06)	0.05 (0.04)		Website	5 (6)	3 (1)	
Implementation monitoring			> .05	Other	37 (46)	45 (13)	
Yes	61 (71)	76 (22)		Provider characteristics	% (*n*) ^a^		> .05
No	4 (5)	7 (2)		Typical provider ^c^			
Not reported	35 (40)	17 (5)		General practitioner (non-primary provider)	10 (12)	7 (2)	
Implementation problems			> .05	Primary care provider	19 (23)	21 (6)	
Yes	13.8 (16)	3 (1)		Behavioral specialist	28 (34)	38 (11)	
Possible	18.1 (21)	21 (6)		Other specialist provider	2 (2)	0 (0)	
Not reported	68.1 (79)	76 (22)		Peer	7 (9)	7 (2)	
Intention-to-treat analysis			> .05	Graduate student/trainee	2 (2)	3 (1)	
Yes	51 (59)	84 (18)		Other provider	33 (41)	24 (7)	
Possible	23 (26)	28 (8)					
No	26 (30)	10 (3)					
CONSORT diagram			> .05				
Yes	82 (94)	90 (26)					
No	18 (20)	10 (3)					
Study and design characteristics	% (*n*) ^a^			Provider characteristics	% (*n*) ^a^		
Overall risk of bias			> .05	Provider profession ^c^			> .05
Unclear	75 (87)	90 (26)		Medical doctor	21 (26)	21 (6)	
High	25 (29)	10 (3)		Physician’s assistant	1 (1)	0 (0)	
Participant characteristics	*M* (*SD*) ^f^			Nurse	11 (14)	7 (2)	
Average age ^c^	34.1 (12.4)	32.6 (13.5)	> .05	Other medical specialist	2 (2)	3 (1)	
Sample age group, % (*n*)			> .05	Psychologist	8 (10)	17 (5)	
Adolescent/young adult	24.1 (28)	31.0 (9)		Social worker	6 (8)	7 (2)	
Mixed or adult only	75.9 (88)	69.0 (20)		Other behavioral health specialist	13 (16)	45 (13)	
Percent female composition ^c^	38.7 (22.7)	42.4 (19.7)	> .05				
Race/ethnicity composition ^c^			> .05				
Percent Asian	13.9 (30.9)	18.3 (40.0)					
Percent Black	28.9 (25.0)	41.3 (27.9)					
Percent Latinx	24.2 (27.2)	38.2 (34.3)					
Percent White	59.9 (27.6)	41.8 (31.4)					

*k* = number of studies. *AD* aggregate data, *IPD* individual participant data

*p*-value reflects statistical significance of comparisons

^a^Percentages and counts shown unless otherwise indicated

^b^Estimates calculated at study level

^c^Estimates calculated at intervention or comparison group level, as appropriate

^d^Number (No.) of boosters calculated only among studies delivering boosters

^e^Interventions could use multiple components; percentages reflect proportion of all intervention groups using each component

^f^Means and standard deviations shown unless otherwise indicated

^g^Chi-square or *t-*tests used to compare the AD and IPD sample

Sociodemographic characteristics were collected at baseline for all trials but were not reported consistently across trials. Most participants were adults (26 or older; *n* = 5,645, 46.8%), followed by young adults (18–25 years; *n* = 2,175, 18%). Only a small portion of the sample were adolescents (*n* = 487, 4%). For those participants who reported sex, 48.9% reported being male (*n* = 5,903) and 38.7% (*n* = 4,675) reported being female. Among participants who had other sociodemographic data available, the majority of participants reported stable housing (*n* = 749, 6.2%), being unemployed (*n* = 1,461, 12.1%), a single relationship status (*n* = 4,509, 37.3%), and having a high school education or above (*n* = 4,036, 33.4%). Among participants who had available baseline severity of substance use data, the majority of participants reported low baseline severity of substance use (*n* = 1,377, 11.4%) followed by moderate use (*n* = 824, 6.8%) and then high use (*n* = 296, 2.5%). Due to inconsistency in reporting of race across primary studies, an adequate representation of all racial and ethnic groups was not possible. Rather, race was dichotomized as non-White and White; 3,238 participants reported being part of non-White racial and ethnic groups (26.8%) and 3,344 participants reported being White (37.7%). The sociodemographic characteristics of the pooled sample are presented in Supplement 7, Table 16; characteristics are also presented by treatment and control conditions, and the proportion of missing data for each of the variables is included. All conclusions about statistical significance below are after correcting for multiple comparisons. Model results corresponding to forest plots are presented in Supplemental Material 7.

### Primary Domains

#### Binge Alcohol Use

**Fig. 2:**
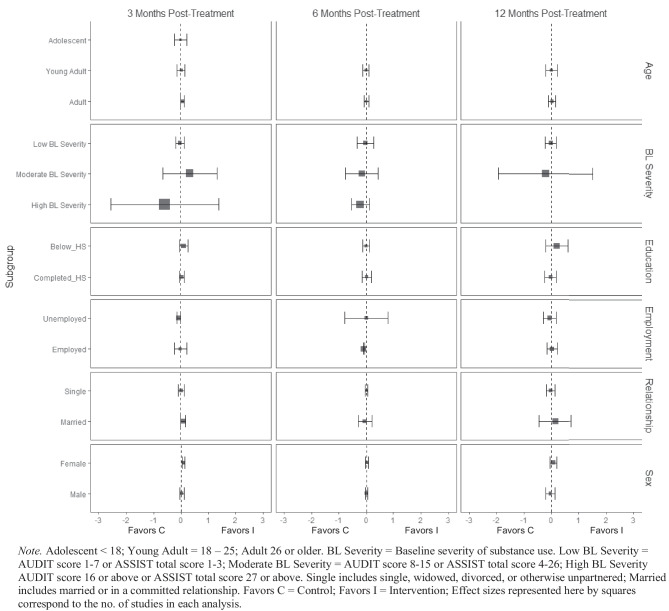
Marginal mean subgroup difference in binge alcohol use shown with 95% confidence intervals

For alcohol consumption at 3 months post-treatment, BIs led to significant reductions in heavy episodic (hereafter referred to as binge) alcohol consumption for females ($\overline{g }$ = 0.09, 95% CI [0.03, 0.14]; *k* = 10), whereas no beneficial effect was observed for males (see Fig. 2,^3^). Heterogeneity across effects was minimal, suggesting that comparable effects for females could be expected in future similar trials (95% PI_*g*_ [0.03, 0.14]).

#### Frequency of Alcohol Use

**Fig. 3:**
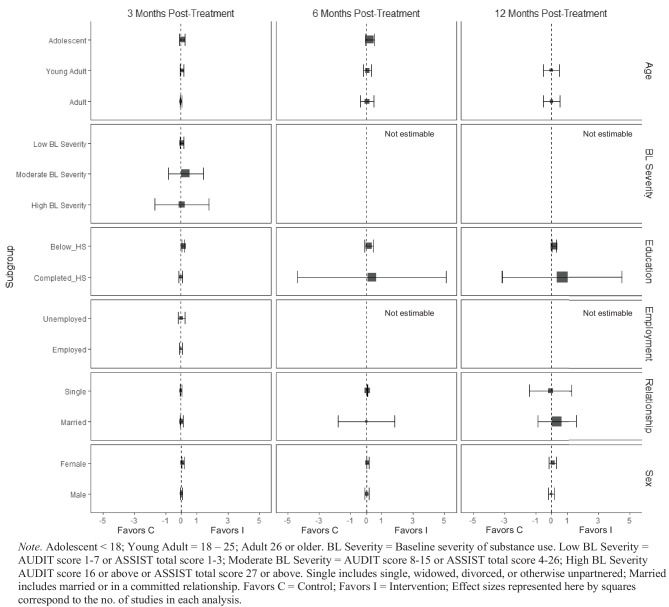
Marginal mean subgroup difference in frequency alcohol use shown with 95% confidence intervals

BIs lead to significant reductions in frequency of alcohol consumption among females ($\overline{g }$ = 0.10, 95% CI [0.03, 0.17]; *k* = 11) (see Fig. 3); heterogeneity across effects was minimal suggesting that comparable beneficial effects for females could be expected in future similar trials (95% PI_*g*_ [0.03, 0.17]). Similarly, results showed that BIs led to significant reductions in frequency of alcohol consumption at 3 months follow-up for individuals with below high school levels of education ($\overline{g }$ = 0.16, 95% CI [0.09, 0.22]; *k* = 6). Heterogeneity in effects was minimal suggesting that similar beneficial effects for individuals with education levels below high school could be expected in future comparable trials (95% PI_*g*_ [0.09, 0.22]).

#### Quantity of Alcohol Use

**Fig. 4:**
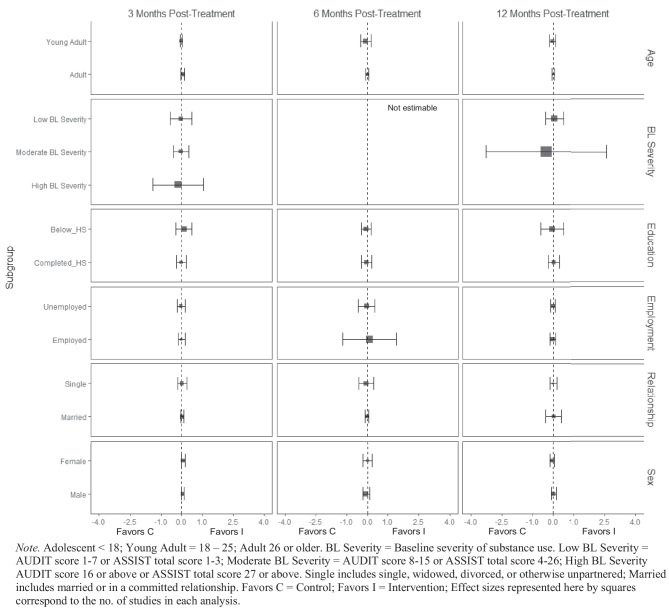
Marginal mean subgroup difference in quantity alcohol use shown with 95% confidence intervals

There was no evidence that BIs led to statistically significant reductions in the quantity of alcohol use for any subgroup moderator of interest at any follow-up assessment period (see Fig. 4).

#### Cannabis, Tobacco, and Other Drug Use

**Fig. 5:**
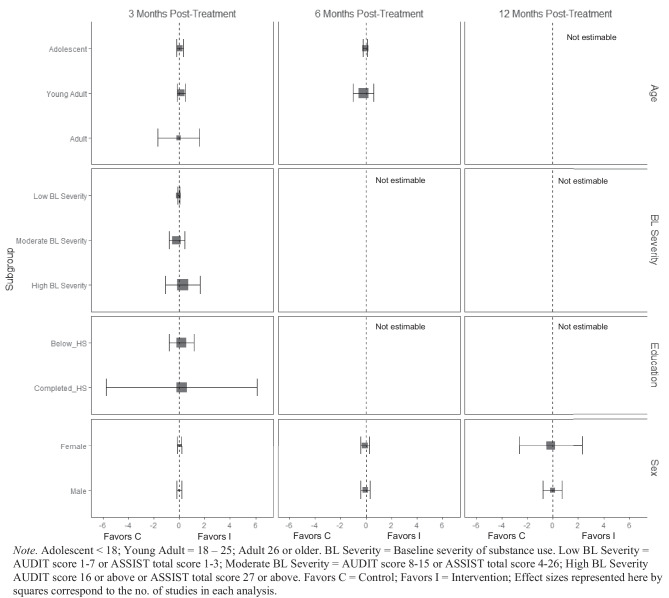
Marginal mean subgroup difference in frequency cannabis use shown with 95% confidence intervals

**Fig. 6:**
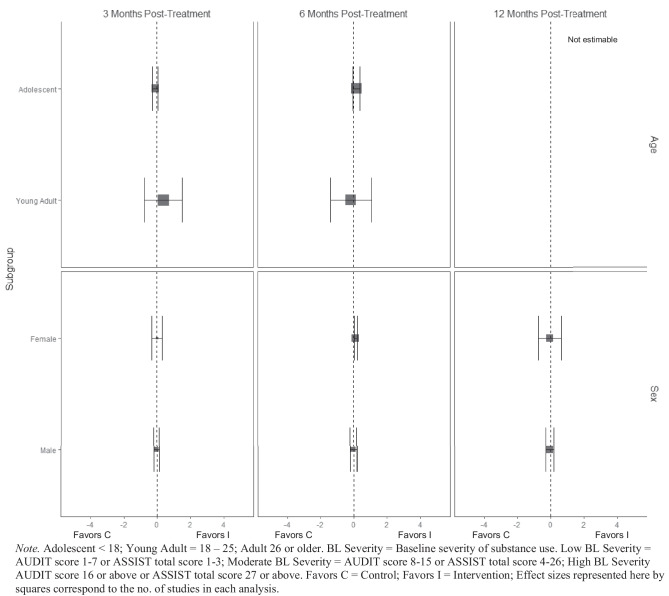
Marginal mean subgroup difference in quantity cannabis use shown with 95% confidence intervals

**Fig. 7:**
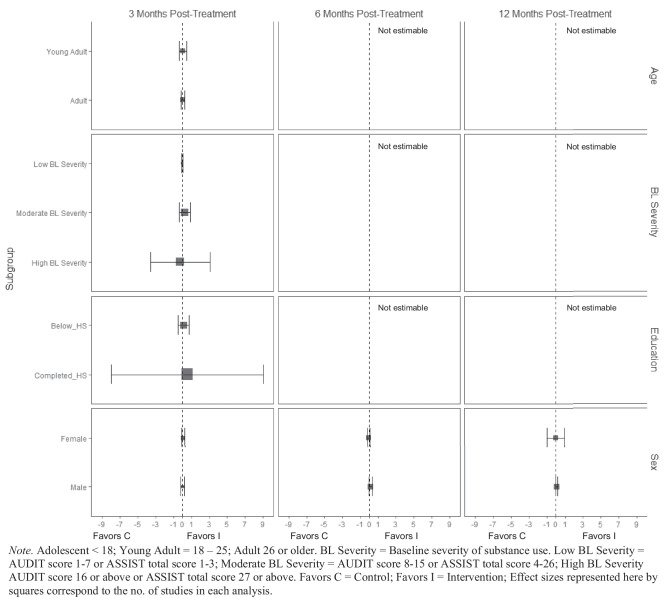
Marginal mean subgroup difference in tobacco use shown with 95% confidence intervals

**Fig. 8:**
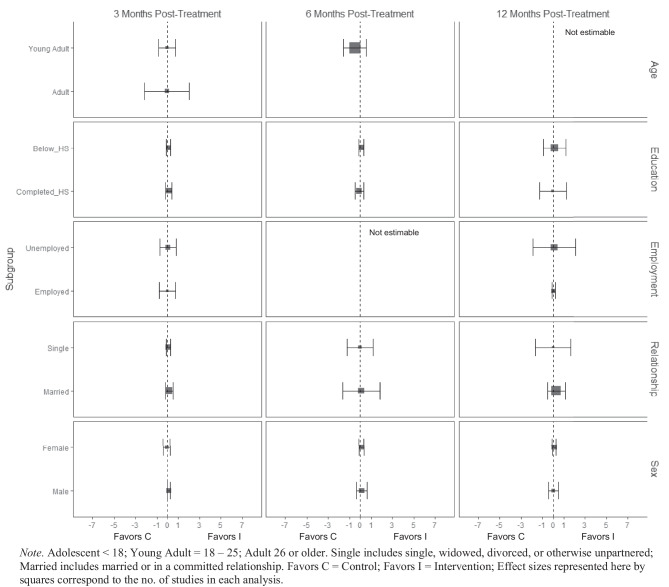
Marginal mean subgroup difference in other drug use shown with 95% confidence intervals

There was no evidence of BIs resulting in beneficial effects for any subgroup of interest for the frequency of cannabis consumption (Fig. 5), and quantity of cannabis consumption outcomes at any 3-, 6-, or 12-month timepoint (Fig. 6), although results should be interpreted with caution given that there were few trials that contributed data (*k* ranged from 2 to 5). No evidence of significant marginal mean subgroup effects at 3 months, 6 months, or 12 months post-treatment for any tobacco use consumption outcome was identified (Fig. 7). Again, these results should be interpreted with caution given the small number of studies contributing effect sizes for these analyses (*k* ranged from 2 to 6). Last, there was no evidence of significant marginal mean subgroup effects found at 3 months, 6 months, or 12 months post-treatment for other drug use consumption (Fig. 8).

#### Alcohol and Other Drug Consequences

**Fig. 9:**
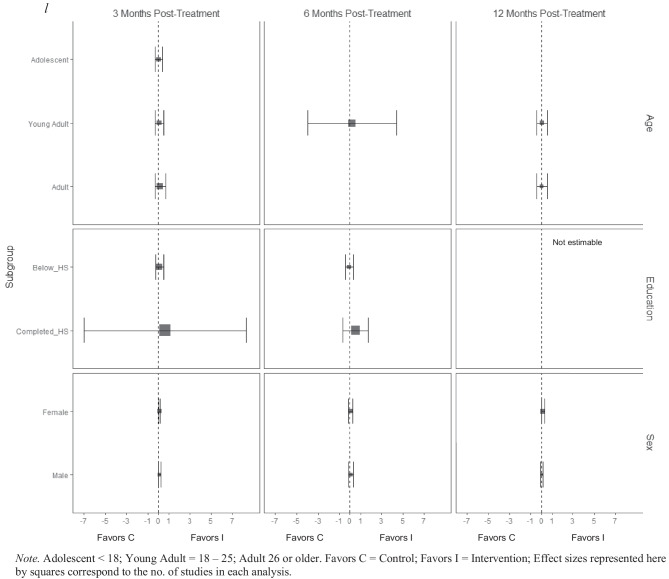
Marginal mean subgroup difference in alcohol-related consequences shown with 95% confidence intervals

**Fig. 10:**
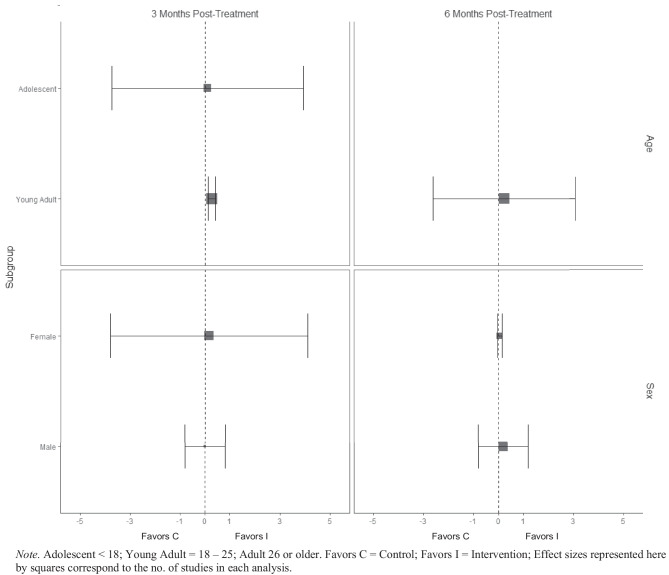
Marginal mean subgroup difference in drug-related consequences shown with 95% confidence intervals

At 3 months post-treatment, BIs led to significant reductions in alcohol-related consequences for females ($\overline{g }$ = 0.16, 95% CI [0.08, 0.25]; *k* = 7), but not for males (see Fig. 9). Heterogeneity across effects was minimal suggesting that similar beneficial effects for females could be expected in future similar trials (95% PI_*g*_ [0.08, 0.25]). No evidence of statistically significant marginal mean subgroup effects was found at 3 months, 6 months, or 12 months post-treatment for other drug-related consequences (Fig. 10).

### Secondary Domains

#### Mental and Physical Health

**Fig. 11:**
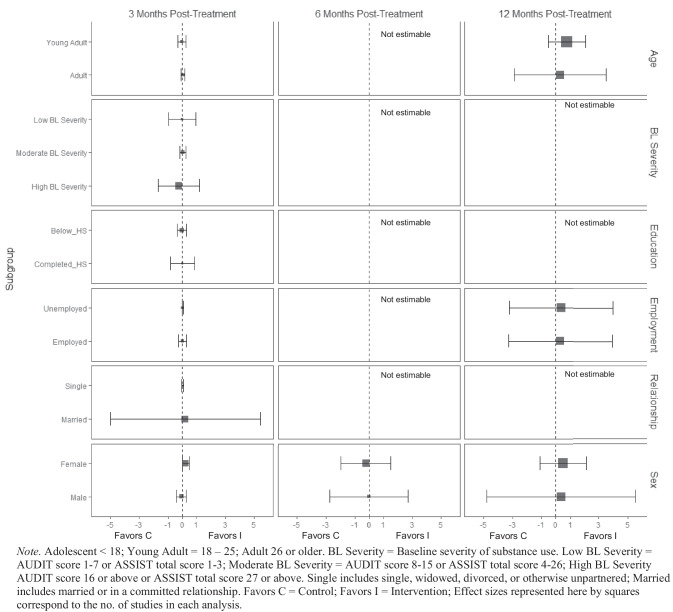
Marginal mean subgroup difference in mental health symptoms shown with 95% confidence intervals

**Fig. 12:**
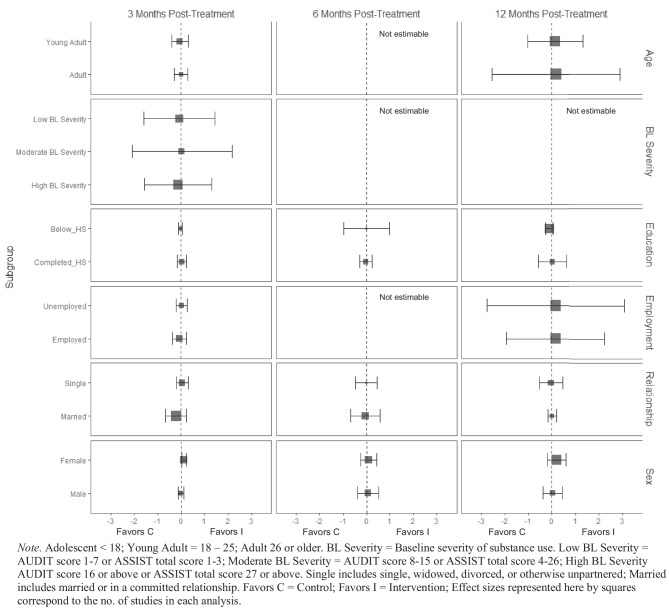
Marginal mean subgroup difference in physical health symptoms shown with 95% confidence intervals

There was no evidence that BIs were associated with statistically significant reductions in mental health symptoms (see Fig. 11) or physical health symptoms (Fig. 12), though these results should be interpreted with caution given the small number of studies contributing data to these analyses (*k* ranged from 2 to 4).

#### Service Utilization

**Fig. 13:**
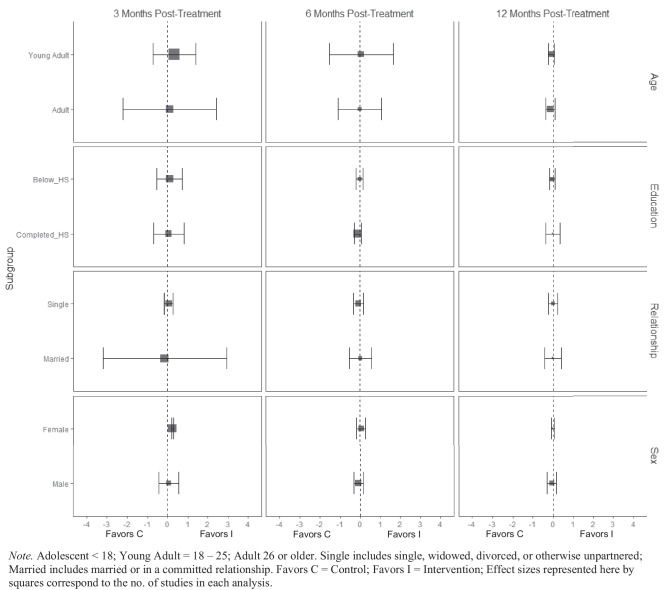
Marginal mean subgroup difference in substance use service utilization shown with 95% confidence intervals

**Fig. 14:**
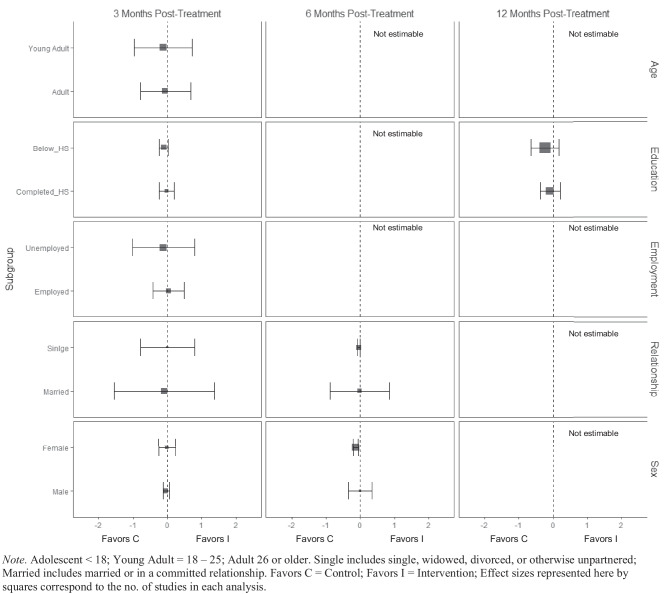
Marginal mean subgroup difference in emergency department utilization shown with 95% confidence intervals

BIs led to greater substance use treatment utilization at 3 months post-treatment for females ($\overline{g }$ = 0.25, 95% CI [0.21, 0.30]; *k* = 3; see Fig. 13) but not males. Heterogeneity across effects was minimal suggesting that similar beneficial effects for females could be expected in future similar trials (95% PI_*g*_ [0.11, 0.39]). There was no evidence that BIs were associated with statistically significant reductions in emergency department use (see Fig. 14).

#### Readiness to Change

**Fig. 15:**
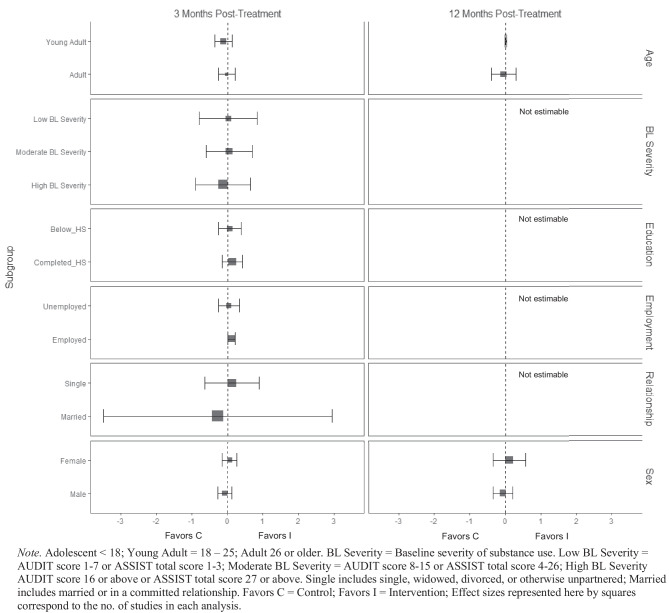
Marginal mean subgroup difference in readiness to change shown with 95% confidence intervals

No evidence of significant marginal mean subgroup effects was found at 3 months, 6 months, or 12 months post-treatment for readiness to change (Fig. 15). These results should be interpreted with caution given the small number of studies contributing effect sizes for these analyses (*k* ranged from 2 to 6).

## Discussion

This IPD meta-analysis synthesized findings from 29 trials and 12,074 individual participants, drawn from a larger AD meta-analysis (Tanner-Smith et al., 2021), with the aim of examining key participant characteristics as moderators of the effects of BIs delivered in general healthcare settings on alcohol and other drug use and healthcare utilization, readiness to change, and physical and mental health. Among primary outcomes, there was evidence that BIs led to reductions in alcohol consumption and alcohol-related consequences and increases in substance use treatment utilization, with larger effects occurring for females and individuals with below high school levels of education at 3 months post-intervention. Despite these differences, the absolute effect magnitudes were small. We also found limited beneficial effects of BIs on other drug outcomes or consequences, which is consistent with prior primary research that found few studies (with inconsistent findings) on the efficacy of drug BIs. Overall, there was limited data available on the effects of BIs on tobacco use and substance use treatment utilization, emergency department utilization, physical and mental health outcomes, and readiness to change.

In this synthesis, findings showed that females have larger intervention-driven reductions in alcohol consumption outcomes in the first 3 months following a BI, including greater reductions in binge alcohol consumption and greater reductions in the frequency of use (e.g., number of drinks in the past week or month) relative to control. Further, there was evidence documenting that females also experienced fewer alcohol-related consequences and greater utilization of substance use treatment following receipt of BIs. These findings contrast with a prior AD meta-analysis that found men have larger BI-driven reductions in alcohol consumption relative to females (Kaner et al., 2018). It is possible that the disparity in sex-moderation findings is due to aggregation bias (i.e., examining the proportion of female participants in primary studies as a moderator of BI effects versus examining interactions between sex and treatment condition at the individual level, as was done in this IPD study). Findings also diverge from an AD meta-analysis documenting that BIs do not increase receipt of alcohol-related services (Glass et al., 2015), whereas the current study suggests females may show increased utilization of substance-related (i.e., both alcohol and other drug) treatment services following BI. These differing findings are less surprising when considering that prior reviews also suggest there are a limited number of studies that have data disaggregated by sex and a larger number of primary studies that focus on BI effects for men only (Kaner et al., 2018; Moyer et al., 2002; O’Donnell et al., 2014). Future research should clarify whether there are additional factors that may drive the sex-specific effects documented in this study. For example, Blow and colleagues (2006) document that younger women, in particular, show reduced heavy episodic drinking following receipt of BI. Further, a prior systematic review suggests that pregnant women who endorse abstinence from alcohol use as a goal when enrolling in BI or women who enroll in BI with heavier drinking profiles may show greater BI-driven abstinence from alcohol consumption (Gilinsky et al., 2011). Future studies might consider these interactions between sex, age, and other goals for abstinence or substance use consumption (e.g., reproductive status) to further the field’s understanding of the profiles of and conditions under which women are most likely to benefit from BI receipt.

Our findings also provide evidence that adults with below high school levels of education may particularly benefit from short-term BI-driven reductions in alcohol consumption. Although there is considerable research documenting the association between greater educational attainment and lowered risk substance use or alcohol or other drug dependence (Bloomfield et al., 2006; Daniel et al., 2009; Paljärvi et al., 2013), this relationship is undoubtedly complex and bidirectional, as illustrated by longitudinal studies on the topic (e.g., Fergusson et al., 2008). Further, there are few studies that directly examine the moderating role of educational attainment on BI or other substance-related program effectiveness (in contrast to research on the effects of educational attainment on relapse, substance use treatment enrollment, or program completion, for which there is some evidence). It should be noted that in the current study, adolescents were not included in the education moderator analyses due to potential confounding with age and because, in general, it would not have been possible for adolescents to have yet obtained a high school diploma. Further, in the current study, only 50% of participants across trials had educational attainment data available; therefore, these results should be interpreted with caution.

The findings from this IPD meta-analysis should be interpreted with several limitations in mind. For several patient characteristics (i.e., effect size moderators), data were not available at the primary study level or were otherwise collapsed or dichotomized into categories that were not able to be harmonized across the 29 primary studies. For example, contextual factors known to exacerbate risk for substance use, including housing status, were only reported in two primary studies. Further, baseline severity of substance use was also inconsistently measured and reported and therefore, nearly 80% of individual participants were missing data on this characteristic. Due to the challenges of data availability and harmonizing variables consistently across primary studies, we made the decision to collapse many participant-level moderator categories to facilitate the inclusion of the greatest number of studies in each analysis. We recognize that this approach does not reflect the true range of individuals’ identities or experiences (e.g., relationship status as “Single” or “Married”). Further, in this study, only one treatment-covariate interaction was included in each model, limiting our ability to further disentangle three-way interactions (e.g., age × employment status × treatment status).

The aim of traditional systematic reviews (employing AD) is typically to identify all available studies relevant to its topic, which in turn yields conclusions based on the complete or near-complete body of evidence. Although it would be optimal for IPD meta-analyses to achieve the same end, many systemic barriers to data sharing exist (Tan et al., 2021), and these exacerbate other issues like the sufficient recruitment, enrollment, and reporting of members of important subgroups. Such barriers are among several commonly faced by researchers conducting IPD meta-analysis that impact the availability of IPD; in our case, these obstacles resulted in an analytic sample comprising one quarter of the studies in the parent AD meta-analysis described above.^4^ We recommend that institutions and funders allocate resources for initiatives that promote the practice of data sharing. These systems-level movements have the potential to facilitate data transparency and promote a more complete, nuanced, and clinically meaningful understanding of how BI and other intervention effects may vary according to participant characteristics. Short of conclusions resting on the “full” body of evidence, however, findings of IPD meta-analyses are based on a larger amount of data than any one study. Consequently, we argue that findings from IPD meta-analyses should not be gauged by the extent to which they represent all available studies on a topic, but instead by whether the studies that are included provide accurate and meaningful estimates of the moderation effects of interest.

Alcohol and other drug use continues to be a public health crisis with profound effects on individuals, families, and communities. The breadth of research on BIs directed at interrupting substance use problems and the negative sequalae that follow demonstrates the field’s commitment to supporting individuals affected by substance use challenges. The current study contributes to a more nuanced understanding of the impact of BIs delivered in general healthcare settings by identifying specific patient groups that may benefit more (or less) from this type of intervention. In light of evidence demonstrating modest BI effects on alcohol use and mixed or null findings for BI effects on other drug use, BI research should continue to investigate potential drivers of effect magnitude and variation. Although the significant BI effects on alcohol use were modest, it is possible that these small effects may be clinically meaningful when evaluated at the population level; therefore, along with others (e.g., Heather, 2012), we suggest researchers consider efforts to examine the population-level effects of BIs in settings where BIs are already widely implemented to determine if BIs result in reductions in population level harms (e.g., rates of alcohol-related illness, injury, or death). Further, even though results show that BIs may lead to modest reductions in alcohol use, it could be that even those small effects are important in interrupting a trajectory of substance use that could be unhealthy, thereby shifting an individual’s substance use from an unhealthier threshold to one that places them at lower risk for negative substance use harms. Researchers should also continue to test novel interventions and program adaptations that could more effectively address substance use for populations for whom BIs demonstrate some evidence of benefit.

### Supplementary Information

Below is the link to the electronic supplementary material.

Supplementary file1 (DOCX 22 KB)

Supplementary file2 (DOCX 25 KB)

Supplementary file3 (DOCX 51 KB)

Supplementary file4 (DOCX 25 KB)

Supplementary file5 (DOCX 40 KB)

Supplementary file6 (DOCX 27 KB)

Supplementary file7 (DOCX 140 KB)

Supplementary file8 (DOCX 87 KB)

## Data Availability

Data were obtained through a restricted data use agreement with primary study authors. Data are not publicly available.

## Footnotes

1 Data inquiries were made with three primary study investigators. Reasons for data queries were: (1) requesting a clustering variable relevant to the study design; (2) clarifying translation and variable accuracy for data provided in a language that was not English; (3) requesting the randomization variable and subsequently verifying original sample size and group baseline characteristics.

2 We planned to examine participant race/ethnicity as a potential moderator (see pre-registered analysis protocol). However, reporting of race was inconsistent across studies and would have necessitated dichotomizing participants into white or non-white subgroups. We judged this approach to be incompatible with our aims of providing finer-grained understanding of BI effects, and ultimately decided against including such a crude race variable among the final set of candidate moderators.

3 For Figs. 2, 3, 4, 5, 6, 7, 8, 9, 10, 11, 12, 13, 14, and 15 referenced throughout the results section, caution should be used when visually comparing effect size estimates across figures and study domains as x-axis are not consistent across figures. See the online supplement for the complete presentation of all numerical results presented in the following paragraphs.

4 Though this is the case, the descriptive comparison between the AD and IPD samples presented in Table 1 show that characteristics of primary studies providing IPD are generally comparable to the full sample of studies in the AD parent meta-analysis (See online supplement; Tanner-Smith et al., 2021).
