# Supplementary material for: Investigating for Whom Brief Substance Use Interventions Are Most Effective: An Individual Participant Data Meta-analysis
Source: Prev Sci. 2023 May 3;24(8):1459–82. doi: 10.1007/s11121-023-01525-1 (PMC10678844; doi:10.1007/s11121-023-01525-1)
Supplement: Supplementary file 1 — Supplementary file1 (DOCX 22 KB) [file 11121_2023_1525_MOESM1_ESM.docx]

**Supplemental Material 1 – PubMed Search Strategy**

(((((((("ambulatory care"[Title/Abstract] OR clinic[Title/Abstract] OR "community care"[Title/Abstract] OR "community health"[Title/Abstract] OR "emergency care"[Title/Abstract] OR "emergency department*"[Title/Abstract] OR "emergency room"[Title/Abstract] OR "family medicine"[Title/Abstract] OR "family physician"[Title/Abstract] OR "family practice"[Title/Abstract] OR "general practice*"[Title/Abstract] OR "general practitioner*"[Title/Abstract] OR "health center"[Title/Abstract] OR hospital[Title/Abstract] OR inpatient[Title/Abstract] OR medical[Title/Abstract] OR nurse[Title/Abstract] OR outpatient[Title/Abstract] OR "patient care"[Title/Abstract] OR physician[Title/Abstract] OR "primary care"[Title/Abstract] OR "primary health care"[Title/Abstract] OR "trauma care"[Title/Abstract] OR "trauma center"[Title/Abstract])) AND ("alcohol reduction"[Title/Abstract] OR "alcohol screening"[Title/Abstract] OR BASICS[Title/Abstract] OR "brief advice"[Title/Abstract] OR "brief alcohol"[Title/Abstract] OR "brief counseling"[Title/Abstract] OR "brief counselling"[Title/Abstract] OR "brief drug"[Title/Abstract] OR "brief intervention"[Title/Abstract] OR "brief physician"[Title/Abstract] OR "brief psychotherapeutic"[Title/Abstract] OR "brief motivation*"[Title/Abstract] OR "brief therapy"[Title/Abstract] OR "brief treatment"[Title/Abstract] OR "early intervention"[Title/Abstract] OR "mailed feedback"[Title/Abstract] OR "minimal intervention"[Title/Abstract] OR "motivational enhancement"[Title/Abstract] OR "motivational intervention"[Title/Abstract] OR "motivational interview*"[Title/Abstract] OR "personalized feedback"[Title/Abstract] OR "personalised feedback"[Title/Abstract] OR SBIRT[Title/Abstract] OR "student assistance program"[Title/Abstract] OR "university assistance program"[Title/Abstract])) AND (alcohol[Title/Abstract] OR amphetamine[Title/Abstract] OR benzodiazepine*[Title/Abstract] OR cannabis[Title/Abstract] OR cocaine[Title/Abstract] OR drink*[Title/Abstract] OR drug[Title/Abstract] OR heroin[Title/Abstract] OR illicit[Title/Abstract] OR marijuana[Title/Abstract] OR opioid[Title/Abstract] OR prescription[Title/Abstract] OR substance[Title/Abstract])))))) AND ("01/01/1989"[PDAT] : "3000"[PDAT]) AND ("1989/01/01"[PDAT] : "3000/12/31"[PDAT])
