# Supplementary material for: Investigating for Whom Brief Substance Use Interventions Are Most Effective: An Individual Participant Data Meta-analysis
Source: Prev Sci. 2023 May 3;24(8):1459–82. doi: 10.1007/s11121-023-01525-1 (PMC10678844; doi:10.1007/s11121-023-01525-1)
Supplement: Supplementary file 2 — Supplementary file2 (DOCX 25 KB) [file 11121_2023_1525_MOESM2_ESM.docx]

Supplemental File 2

Data Harmonization

| **Moderators** | | | |
| --- | --- | --- | --- |
| **Variable** | **Coding** | | **Notes** |
| Sex | 0 = male  1 = female | |  |
| Relationship Status | 0 = single  1 = married | | Married could contain the following categories: “in partnership,” “married,” “stable partnership,” “have a partner”  Single could contain the following categories: “single,” “divorced/separated,” “widowed,” “never married,” “other” |
| Baseline Severity of Use | 0 = low BL severity  1 = moderate BL severity  2 = high BL severity | | Low BL Severity = AUDIT score 1-7 or ASSIST total score 1-3;  Moderate BL Severity = AUDIT score 8-15 or ASSIST total score 4-26;  High BL Severity AUDIT score 16 or above or ASSIST total score 27 or above |
| Education | 0 = below high school level of education  1 = high school degree or above | | Adolescent participants as defined in this study (< 18 years of age) were not included in analyses involving the education moderator due to them not having the ability to achieve a high school education |
| Employment Status | 0 = unemployed  1 = employed | | Employed could have contained “employed part-time,” “self-employed,” “employed full-time,” “economically active,” “worked in the past year”  Unemployed could have contained “retired,” or “other,” “unemployed and not looking for work,” “unemployed and looking for work,” “homemaker/caring for children,” “sick leave, disabled,” “temporarily laid off,” “economically inactive.” |
| Housing | 0 = currently unhoused  1 = stable housing | | Currently unhoused could have contained “homeless in past 3 months” |
| Age | 0 = Adolescent = < 18  1 = Young Adult = 18-25  2 = Adult =< 26 | | Adolescent = < 18  Young Adult = 18-25  Adult =< 26 |
| **Outcome Domains** | | | |
| **Domain** | | **Defined** | |
| Frequency alcohol consumption | | Any measure pertaining to the frequency of alcohol consumption, for example, number of days drinking in the past week or month | |
| Quantity alcohol consumption | | Any measure pertaining to the quantity of alcohol consumption within a drinking occasion, for example, the number of drinks in the past 7 days, or past 30 days | |
| Binge alcohol consumption | | Any measure pertaining to the presence of binge alcohol consumption, for example, number of times you have drank more than four drinks in one drinking occasion, number of binges in the past 30 days | |
| Alcohol-related consequences | | Any measure pertaining to consequences from alcohol use specifically | |
| Tobacco consumption | | Any measure of frequency or quantity of tobacco consumption | |
| Frequency cannabis consumption | | Any measure pertaining to the frequency of cannabis consumption, for example, number of days using cannabis in the past week or month | |
| Quantity cannabis consumption | | Any measure pertaining to the quantity of cannabis consumption | |
| Cocaine/opioids/methamphetamine/mixed/other illicit drug consumption | | Any measure of frequency or quantity of other drugs not including alcohol, cannabis, or tobacco, including nonprescription use of prescription medications | |
| Drug-related consequences | | Any measure pertaining to consequences from drug use not including alcohol use | |
| Mental health symptoms | | Global or specific measures of mental health | |
| Physical health symptoms | | Global measures of physical health | |
| Substance use treatment utilization | | Any measure of the presence of substance use treatment utilization following randomization or the number of days spent in substance use treatment utilization. These measures could be inpatient our outpatient substance use treatment. | |
| Emergency department utilization | | Any measure of emergency department use related to substance use or the number of days spent in the emergency department due to substance use reasons | |
| Readiness to change | | Motivation measures that assessed readiness to change substance use behaviors specifically. | |
