# Supplementary material for: Investigating for Whom Brief Substance Use Interventions Are Most Effective: An Individual Participant Data Meta-analysis
Source: Prev Sci. 2023 May 3;24(8):1459–82. doi: 10.1007/s11121-023-01525-1 (PMC10678844; doi:10.1007/s11121-023-01525-1)
Supplement: Supplementary file 3 — Supplementary file3 (DOCX 51 KB) [file 11121_2023_1525_MOESM3_ESM.docx]

BRIEF SUBSTANCE USE INTERVENTIONS IN GENERAL HEALTHCARE SETTINGS

META-ANALYSIS

**INDIVIDUAL PARTICIPANT DATA ANALYSIS PLAN**

*Version 2: May 2022*

The following analysis plan for the *Brief Substance Use Interventions in General Healthcare Settings*meta-analysis summarizes analyses planned for synthesizing individual participant data (IPD). Detail is provided on 1) individual participant-level data collection and handling, primary and secondary outcomes, and analyses, and 2) synthesizing effects derived from IPD.

**DATA COLLECTION AND CLEANING**

Primary study investigators of all eligible trials were contacted through email with an overview of the proposed IPD meta-analysis and an invitation to collaborate. Following initial contact, primary study investigators were sent a scripted email and offered their preference of either (1) a teleconference with the IPD meta-analysis principal investigator and co-investigators; and/or, (2) an email containing detailed information on the proposed IPD meta-analysis, a templated data sharing/data use agreement, information concerning the de-identification of data and protection of human subjects, and options for the secure transfer of data. In the case of no response, a series of reminder emails were sent every two weeks for two months. If no response was received, the IPD were recorded as unavailable due to investigator non-response. When requested, primary study investigators were provided with a dataset template listing requested variables based on a pre-specified list of moderators and effect sizes central to the IPD meta-analysis study aims, in conjunction with information drawn from primary study registries, protocols, and published reports. When available, IPD from primary studies were acquired directly from open-access data repositories. De-identified IPD provided by primary study investigators were stored on a secure server and accessed only by authorized research study personnel. IPD meta-analysis findings will report information on the proportion of primary studies and primary study participants obtained, and the reasons primary study data were not available. Although publication bias will not be assessed among studies providing IPD because they comprise a non-representative subsample of the studies identified using the project’s overall inclusion criteria, final reports from this study will present descriptive comparisons between aggregate data (AD) studies and studies that contributed IPD in an effort to assess the IPD sample representativeness against the unavailable studies that were included in the project’s corresponding AD meta-analysis. Publication bias will be quantitatively and graphically assessed in the corresponding AD meta-analysis.

Upon receipt of de-identified IPD, data files will be checked to ensure they can be read and loaded into the R environment (R Core Team, 2019; RStudio Team, 2019), where all data cleaning and analysis will be performed. Data will be checked for consistency with published reports, including checking for missing values. Primary study sample size will be checked against published primary study reports, trial registries, and protocols, to confirm all participants that were recruited and randomized were included in each data file. Files will be checked to confirm no obvious omissions or duplicates in the sequence of patient identifiers. Further, data will be checked to detect any inconsistencies in the number of participants per condition given each primary study’s randomization schedule. Data distributions will be checked for any missing, invalid, out of range, or inconsistent values. Baseline equivalence between primary study arms will be checked using all available demographics, including age, race/ethnicity, sex, relationship status, income, housing status, employment, baseline physical and mental health comorbidities, and baseline drug and alcohol use. Data inconsistencies or missing data will be discussed with relevant study investigators and corrected when necessary.

**PRIMARY AND SECONDARY OUTCOMES**

The IPD meta-analysis will focus on the following primary and secondary outcomes:

Primary outcome domains:

1. Alcohol consumption
   - 1. Frequency alcohol consumption
     2. Quantity alcohol consumption
2. Alcohol-related consequences
3. Tobacco consumption
4. Cannabis consumption

i) Frequency cannabis consumption

ii) Quantity cannabis consumption

1. Cocaine/opioids/methamphetamine/mixed/other illicit drug consumption

6. Drug-related consequences

7. Binge Alcohol Use

Secondary outcome domains:

1. Mental health symptoms
2. Physical health symptoms

3. Substance use treatment utilization

4. Emergency department utilization

1. Readiness to change

* Additional primary outcome domains highlighted above were added given the shift in analytic strategy and need to ensure the statistical independence of effect sizes from each primary study.

**MAIN ANALYSES**

Synthesis of IPD will be carried out using a two-stage modelling approach (e.g., Burke et al., 2017; Stewart et al., 2012): In the first stage, intervention effects and intervention by covariate interaction effects will be estimated at the primary study level; in the second stage, those effects will be pooled across all studies using standard meta-analysis methods. Prior to conducting the first stage of analysis, missing data will be multiply imputed at the primary study level using all available data to recover variable values and uncertainty associated with sampling, measurement, and imputation. Prior to imputation, all predictor and outcome variables will be standardized so that model output is in the form of standardized regression coefficients. Interaction (moderation) effects will be the effect of interest during the first stage of analysis (because estimation of overall intervention effectiveness will be based on the project’s corresponding AD meta-analysis). In all primary study-level outcomes models, linear or logistic regression will be used as appropriate, with pretest values of outcomes included to adjust for baseline values and relevant blocking/stratification/clustering accounted for in the models using Huber-White cluster-robust standard errors.

At each wave of measurement, outcomes in the above primary and secondary outcome domains will be interacted with the following participant level moderators, as available in primary studies:

1. Race/ethnicity (categorical)
2. Sex (male/female)
3. Age (categorical: adolescent, young adult, adult)
4. Co-occurring mental health diagnosis (yes/no)
5. Education level (high school or below vs. above high school)
6. Employment status (employed/unemployed)
7. Housing status (stable/unstable)
8. Relationship status (single/in a relationship)
9. Baseline Use/Severity moderator

* We added baseline severity of substance use as a moderator following feedback provided by the expert consultants on the project. We shifted age to from a continuous variable to a categorical variable that included the three levels of adolescent, young adult, and adult. We made this shift due to a large number of studies submitting age data as a similar categorical variable. We removed the pediatric sample moderator as we included an adolescent age moderator.

*Decision rules for selection of effect sizes from primary study*

Only one effect size per individual participant dataset for each domain (e.g., frequency alcohol consumption, other drug consumption) and at each timepoint (e.g., 3-month, 12-month) was included in each analysis to ensure the statistical independence of the effect size estimates in each analysis (i.e., standardized interaction effects between randomization condition and participant level moderators). For studies that reported multiple outcome effect sizes within domains (for example, weekly and monthly frequency of alcohol use), we used decision rules to select the effect size to be used in the analysis. Specifically, preference was given to effect sizes that were: (1) continuous measures versus dichotomous measures; (2) the more general measure (i.e., general mental health versus depression or anxiety scores); (3) the effect size reported in the time frame that is most commonly represented in the IPD data available across all studies; (4) the effect size that has a corresponding baseline score; and (5) the effect size specific to drug or alcohol use (i.e., an emergency room visit related to substance use versus all hospitalizations). Regarding dependences for studies with multiple treatment contrasts (i.e., control versus screening and assessment; control versus BI), we will select the treatment contrast between the most minimal control group and the most intensive intervention. A sensitivity analysis will be run to assess whether the treatment contrast between the control and lesser intensive intervention (e.g., screening and assessment) substantively alters study findings.

* The decision rules highlighted above were added given the shift in analytic strategy and the need to ensure the statistical independence of effect sizes from each primary study.

In the second stage of analysis, mean effect sizes pooled across studies will be estimated separately for the primary and secondary outcome domains and at each follow-up period. This second stage in the synthesis will use random-effects meta-analysis models due to the anticipated heterogeneity of effects among synthesized studies as well as an interest in generalizing findings. Given the analytic approach described above in stage one, the primary effect size synthesized will be pooled pretest-adjusted standardized marginal subgroup effects (e.g., the subgroup effect estimated from the interaction between the intervention condition and moderator of interest). Random effects inverse variance-weighted mean effect sizes and 95% confidence intervals will be calculated for each analysis. The magnitude of between-study variation or heterogeneity ($\tau^{2}$) will be estimated using a restricted maximum likelihood estimator. Additionally, a Benjamini-Hochberg procedure for multiple comparisons (Benjamini & Hochberg, 1995) will be implemented to control Type I error rates for all analyses conducted within an outcome domain.

All data handling, missing data imputation, and analyses will be carried out with scripted and manual procedures in R. Specific packages utilized will include *mice* (Version 3.3.0; van Buuren & Groothuis-Oudshoorn, 2018) for multiple imputation of missing data, and *metafor* (v2.4-0; Viechtbauer, 2010) for stage-two meta-analysis.

* Due to the use of a shifting unit of analysis approach that ensures no statistically dependent effect sizes will be included in any given meta-analysis, we will no longer use RVE for the second stage of the IPD analysis. We used the package *metafor* (v2.4-0; Viechtbauer, 2010) instead of *robumeta* given the shift in our analytic strategy described above.

**REFERENCES**

Benjamini, Y., & Hochberg, Y. (1995). Controlling the false discovery rate: A practical and powerful approach to multiple testing. *Journal of the Royal Statistical Society*, *57*(1), 289–300. https://doi.org/10.1111/j.2517-6161.1995.tb02031.x

Burke, D. L., Ensor, J., & Riley, R. D. (2017). Meta-analysis using individual participant data: One-stage and two-stage approaches, and why they may differ. *Statistics in Medicine*, *36*(5), 855–875. https://doi.org/10.1002/sim.7141

Fisher, Z., Tipton, E., & Zhipeng, H. (2017). *robumeta: Robust variance meta-regression* (Version 2.0) [Computer software]. https://cran.r-project.org/web/packages/robumeta/index.html

R Core Team. (2019). *R: A language and environment for statistical computing* (Version 3.6.0) [Computer software]. R Foundation for Statistical Computing. https://www.R-project.org/

RStudio Team. (2019). *RStudio: Integrated development environment for R* (Version 1.2.1335) [Computer software]. RStudio, Inc. http://www.rstudio.com/

Stewart, G. B., Altman, D. G., Askie, L. M., Duley, L., Simmonds, M. C., & Stewart, L. A. (2012). Statistical analysis of individual participant data meta-analyses: A comparison of methods and recommendations for practice. *PLoS ONE*, *7*(10), e46042. https://doi.org/10.1371/journal.pone.0046042

van Buuren, S., & Groothuis-Oudshoorn, K. (2018). *MICE: Multivariate imputation by chained equations* (Version 3.3.0) [Computer software]. https://cran.r-project.org/web/packages/mice/mice.pdf

Viechtbauer W (2010). “Conducting meta-analyses in R with the metafor package.” Journal of Statistical Software*, 36*(3), 1–48. <https://doi.org/10.18637/jss.v036.i03>.
