# Supplementary material for: Investigating for Whom Brief Substance Use Interventions Are Most Effective: An Individual Participant Data Meta-analysis
Source: Prev Sci. 2023 May 3;24(8):1459–82. doi: 10.1007/s11121-023-01525-1 (PMC10678844; doi:10.1007/s11121-023-01525-1)
Supplement: Supplementary file 4 — Supplementary file4 (DOCX 25 KB) [file 11121_2023_1525_MOESM4_ESM.docx]

Supplemental Material S3: Results from Sensitivity Analysis

**Method**

A sensitivity analysis was run to assess whether the treatment contrast between the control and lesser intensive intervention (e.g., screening and assessment) substantively altered main study findings. We removed treatment contrasts between control and the more intensive intervention for 8 of the 29 studies listed below. We replaced them with treatment contrasts between control and the lesser intensive intervention, which was often a screening or assessment only condition. See detailed notes in the table below regarding the treatment contrasts included in the main paper versus those included in the sensitivity analysis.

| **Studies with multiple control and treatment contrasts** |
| --- |
| Cherpitel et al., 2009 |
| Cherpitel et al., 2016 |
| Bischof et al., 2008 |
| Rhoades et al., 2015 |
| Heather et al., 2004 |
| Bogenshutz et al., 2014 |
| Daeppen et al., 2007 |
| Saitz et al., 2014 |

**Results**

Results presented here are those focusing on effects that were statistically significant following correction for multiple comparisons through the Benjamini-Hochberg procedure.

When conducting the sensitivity analysis, there were no additional statistically significant effects that were not also presented in the main analyses.

Additionally, all Benjamini-Hochberg corrected statistically significant results in the main analyses remained statistically significant when analyzed in the sensitivity analyses.
