# Supplementary material for: Investigating for Whom Brief Substance Use Interventions Are Most Effective: An Individual Participant Data Meta-analysis
Source: Prev Sci. 2023 May 3;24(8):1459–82. doi: 10.1007/s11121-023-01525-1 (PMC10678844; doi:10.1007/s11121-023-01525-1)
Supplement: Supplementary file 5 — Supplementary file5 (DOCX 40 KB) [file 11121_2023_1525_MOESM5_ESM.docx]

**Supplemental Material 2 – References to Studies Included in Meta-Analysis**

1. Alford, D. P., German, J. S., Samet, J. H., Cheng, D. M., Lloyd-Travaglini, C. A., & Saitz, R. (2016). Primary care patients with drug use report chronic pain and self-medicate with alcohol and other drugs. *Journal of General Internal Medicine*, *31*(5), 486–491. https://doi.org/10.1007/s11606-016-3586-5
2. Arnaud, N., Diestelkamp, S., Wartberg, L., Sack, P., Daubmann, A., & Thomasius, R. (2017). Short‐ to midterm effectiveness of a brief motivational intervention to reduce alcohol use and related for alcohol intoxicated children and adolescents in pediatric emergency departments: A randomized controlled trial. *Academic Emergency Medicine*, *24*(2), 186–200. https://doi.org/10.1111/acem.13126
3. Assanangkornchai, S., Nima, P., McNeil, E. B., & Edwards, J. G. (2015). Comparative trial of the WHO ASSIST-linked brief intervention and simple advice for substance abuse in primary care. *Asian Journal of Psychiatry*, *18*, 75–80. https://doi.org/10.1016/j.ajp.2015.09.003
4. Barticevic, N., Poblete, F., Zuzulich, S., Quevedo, D., & Sena, B. Health technicians provided brief intervention versus minimal intervention for overloaded primary care centers in Chile. A randomized controlled trial. Manuscript in Preparation. 2020.
5. Baumeister, S. E., Gelberg, L., Leake, B. D., Yacenda-Murphy, J., Vahidi, M., & Andersen, R. M. (2014). Effect of a primary care based brief intervention trial among risky drug users on health-related quality of life. *Drug and Alcohol Dependence*, *142*, 254–261. https://doi.org/10.1016/j.drugalcdep.2014.06.034
6. Becker, S. J., Spirito, A., Hernandez, L., Barnett, N. P., Eaton, C. A., Lewander, W., Rohsenow, D. J., & Monti, P. M. (2012). Trajectories of adolescent alcohol use after brief treatment in an Emergency Department. *Drug and Alcohol Dependence*, *125*(1–2), 103–109. https://doi.org/10.1016/j.drugalcdep.2012.03.021
7. Bernstein, J., Bernstein, E., Hudson, D., Belanoff, C., Cabral, H. J., Cherpitel, C. J., Bond, J., Ye, Y., Woolard, R., Villalobos, S., & Ramos, R. (2017). Differences by gender at twelve months in a brief intervention trial among Mexican-origin young adults in the emergency department. *Journal of Ethnicity in Substance Abuse*, *16*(1), 91–108. https://doi.org/10.1080/15332640.2015.1095667
8. Bertholet, N., Palfai, T., Gaume, J., Daeppen, J.-B., & Saitz, R. (2014). Do brief alcohol motivational interventions work like we think they do? *Alcoholism: Clinical and Experimental Research*, *38*(3), 853–859. https://doi.org/10.1111/acer.12274
9. Bischof, G., Freyer-Adam, J., Meyer, C., John, U., & Rumpf, H.-J. (2012). Changes in drinking behavior among control group participants in early intervention studies targeting unhealthy alcohol use recruited in general hospitals and general practices. *Drug and Alcohol Dependence*, *125*(1–2), 81–88. https://doi.org/10.1016/j.drugalcdep.2012.03.018
10. Bischof, G., Grothues, J. M., Reinhardt, S., Meyer, C., John, U., & Rumpf, H.-J. (2008). Evaluation of a telephone-based stepped care intervention for alcohol-related disorders: A randomized controlled trial. *Drug and Alcohol Dependence*, *93*(3), 244–251. https://doi.org/10.1016/j.drugalcdep.2007.10.003
11. Bogenschutz, M. P., Donovan, D. M., Adinoff, B., Crandall, C., Forcehimes, A. A., Lindblad, R., Mandler, R. N., Oden, N. L., Perl, H. I., & Walker, R. (2011). Design of NIDA CTN Protocol 0047: Screening, motivational assessment, referral, and treatment in emergency departments (SMART-ED). *The American Journal of Drug and Alcohol Abuse*, *37*(5), 417–425. https://doi.org/10.3109/00952990.2011.596971
12. Bogenschutz, M. P., Donovan, D. M., Mandler, R. N., Perl, H. I., Forcehimes, A. A., Crandall, C., Lindblad, R., Oden, N. L., Sharma, G., Metsch, L., Lyons, M. S., McCormack, R., Konstantopoulos, W. M., & Douaihy, A. (2014). Brief intervention for patients with problematic drug use presenting in emergency departments: A randomized clinical trial. *JAMA Internal Medicine*, *174*(11), 1736. https://doi.org/10.1001/jamainternmed.2014.4052
13. Bruguera, P., Barrio, P., Oliveras, C., Braddick, F., Gavotti, C., Bruguera, C., López‐Pelayo, H., Miquel, L., Segura, L., Colom, J., Ortega, L., Vieta, E., & Gual, A. (2018). Effectiveness of a specialized brief intervention for at‐risk drinkers in an emergency department: Short‐term results of a randomized controlled rrial. *Academic Emergency Medicine*, *25*(5), 517–525. https://doi.org/10.1111/acem.13384
14. Cancilliere, M. K., Spirito, A., Monti, P., & Barnett, N. (2018a). Brief alcohol interventions for youth in the emergency department: Exploring proximal and distal outcomes. *Journal of Child & Adolescent Substance Abuse*, *27*(5–6), 311–321. https://doi.org/10.1080/1067828X.2018.1529645
15. Cancilliere, M. K., Spirito, A., Monti, P., & Barnett, N. (2018b). Brief alcohol interventions for youth in the emergency department: Exploring proximal and distal outcomes. *Journal of Child & Adolescent Substance Abuse*, *27*(5–6), 311–321. https://doi.org/10.1080/1067828X.2018.1529645
16. Chavez, K. E., Palfai, T. P., Squires, L. E., Cheng, D. M., Lloyd-Travaglini, C., & Saitz, R. (2018). Perceived discrimination and drug involvement among black primary care patients who use drugs. *Addictive Behaviors*, *77*, 63–66. https://doi.org/10.1016/j.addbeh.2017.08.029
17. Cherpitel, C. J., Bernstein, E., Bernstein, J., Moskalewicz, J., & Swiatkiewicz, G. (2009). Screening, brief intervention and referral to treatment (SBIRT) in a Polish emergency room: Challenges in cultural translation of SBIRT. *Journal of Addictions Nursing*, *20*(3), 127–131. https://doi.org/10.1080/10884600903047618
18. Cherpitel, C. J., Korcha, R. A., Moskalewicz, J., Swiatkiewicz, G., Ye, Y., & Bond, J. (2010). Screening, brief intervention, and referral to treatment (SBIRT): 12-Month outcomes of a randomized controlled clinical trial in a Polish emergency department: *Alcoholism: Clinical and Experimental Research*, *34*(11), 1922–1928. https://doi.org/10.1111/j.1530-0277.2010.01281.x
19. Cherpitel, C. J., Moskalewicz, J., Swiatkiewicz, G., Ye, Y., & Bond, J. (2009). Screening, brief intervention, and referral to treatment (SBIRT) in a Polish emergency department: Three-month outcomes of a randomized, controlled clinical trial. *Journal of Studies on Alcohol and Drugs*, *70*(6), 982–990. https://doi.org/10.15288/jsad.2009.70.982
20. Cherpitel, C. J., Ye, Y., Bond, J., Woolard, R., Villalobos, S., Bernstein, J., Bernstein, E., & Ramos, R. (2016). Brief intervention in the emergency department among Mexican-origin young adults at the US–Mexico border: Outcomes of a randomized controlled clinical trial using promotores. *Alcohol and Alcoholism*, *51*(2), 154–163. https://doi.org/10.1093/alcalc/agv084
21. Cherpitel, C. J., Ye, Y., Moskalewicz, J., & Świątkiewicz, G. (2015). Does brief intervention work for heavy episodic drinking? A comparison of emergency department patients in two cultures. *Alcoholism and Drug Addiction*, *28*(3), 145–162. https://doi.org/10.1016/j.alkona.2015.05.001
22. Daeppen, J.-B., Bertholet, N., & Gaume, J. (2010). What process research tells us about brief intervention efficacy: Brief intervention efficacy. *Drug and Alcohol Review*, *29*(6), 612–616. https://doi.org/10.1111/j.1465-3362.2010.00235.x
23. Daeppen, J.-B., Bertholet, N., Gmel, G., & Gaume, J. (2007). Communication during brief intervention, intention to change, and outcome. *Substance Abuse*, *28*(3), 43–51. https://doi.org/10.1300/J465v28n03_05
24. Daeppen, J.-B., Gaume, J., Bady, P., Yersin, B., Calmes, J.-M., Givel, J.-C., & Gmel, G. (2007). Brief alcohol intervention and alcohol assessment do not influence alcohol use in injured patients treated in the emergency department: A randomized controlled clinical trial. *Addiction*, *102*(8), 1224–1233. https://doi.org/10.1111/j.1360-0443.2007.01869.x
25. D’Amico, E. J., Miles, J. N. V., Stern, S. A., & Meredith, L. S. (2008). Brief motivational interviewing for teens at risk of substance use consequences: A randomized pilot study in a primary care clinic. *Journal of Substance Abuse Treatment*, *35*(1), 53–61. https://doi.org/10.1016/j.jsat.2007.08.008
26. D’Amico, E. J., Parast, L., Osilla, K. C., Seelam, R., Meredith, L. S., Shadel, W. G., & Stein, B. D. (2019). Understanding which teenagers benefit most from a brief primary care substance use intervention. *Pediatrics*, *144*(2), e20183014. https://doi.org/10.1542/peds.2018-3014
27. D’Amico, E. J., Parast, L., Shadel, W. G., Meredith, L. S., Seelam, R., & Stein, B. D. (2018). Brief motivational interviewing intervention to reduce alcohol and marijuana use for at-risk adolescents in primary care. *Journal of Consulting and Clinical Psychology*, *86*(9), 775–786. https://doi.org/10.1037/ccp0000332
28. Diestelkamp, S., Wartberg, L., Arnaud, N., & Thomasius, R. (2016). Einfluss von Berater/innen- und Interventionsvariablen auf die Veränderungsmotivation nach einer motivierenden Kurzintervention zur Reduktion riskanten Alkoholkonsums. *Praxis der Kinderpsychologie und Kinderpsychiatrie*, *65*(7), 534–549. https://doi.org/10.13109/prkk.2016.65.7.534
29. Donovan, D. M., Bogenschutz, M. P., Perl, H., Forcehimes, A., Adinoff, B., Mandler, R., Oden, N., & Walker, R. (2012). Study design to examine the potential role of assessment reactivity in the Screening, Motivational Assessment, Referral, and Treatment in Emergency Departments (SMART-ED) protocol. *Addiction Science & Clinical Practice*, *7*(1), 16. https://doi.org/10.1186/1940-0640-7-16
30. Forcehimes, A. A., Bogenschutz, M., Sharma, G., & Mandler, R. (2015). Race and ethnicity differences in a MI-based brief intervention delivered in an ED setting. *Drug and Alcohol Dependence*, *146*, e280–e281. https://doi.org/10.1016/j.drugalcdep.2014.09.229
31. Fuster, D., Cheng, D. M., Wang, N., Bernstein, J. A., Palfai, T. P., Alford, D. P., Samet, J. H., & Saitz, R. (2016). Brief intervention for daily marijuana users identified by screening in primary care: A subgroup analysis of the ASPIRE randomized clinical trial. *Substance Abuse*, *37*(2), 336–342. https://doi.org/10.1080/08897077.2015.1075932
32. Gelberg, L., Andersen, R. M., Afifi, A. A., Leake, B. D., Arangua, L., Vahidi, M., Singleton, K., Yacenda‐Murphy, J., Shoptaw, S., Fleming, M. F., & Baumeister, S. E. (2015). Project QUIT (Quit Using Drugs Intervention Trial): A randomized controlled trial of a primary care‐based multi‐component brief intervention to reduce risky drug use. *Addiction*, *110*(11), 1777–1790. https://doi.org/10.1111/add.12993
33. Gelberg, L., Andersen, R. M., Rico, M. W., Vahidi, M., Natera Rey, G., Shoptaw, S., Leake, B. D., Serota, M., Singleton, K., & Baumeister, S. E. (2017). A pilot replication of QUIT, a randomized controlled trial of a brief intervention for reducing risky drug use, among Latino primary care patients. *Drug and Alcohol Dependence*, *179*, 433–440. https://doi.org/10.1016/j.drugalcdep.2017.04.022
34. Gelberg, L., Andersen, R., Vahidi, M., Rico, M., Baumeister, S., & Leake, B. (2017). A multi-component brief intervention for risky drug use among Latino patients of a federally qualified health center in East Los Angeles: A randomized controlled trial of the quit using drugs intervention trial (QUIT) brief intervention. *Drug and Alcohol Dependence*, *171*, e70–e71. https://doi.org/10.1016/j.drugalcdep.2016.08.203
35. Grothues, J. M., Bischof, G., Reinhardt, S., Meyer, C., John, U., & Rumpf, H.-J. (2008). Effectiveness of brief alcohol interventions for general practice patients with problematic drinking behavior and comorbid anxiety or depressive disorders. *Drug and Alcohol Dependence*, *94*(1–3), 214–220. https://doi.org/10.1016/j.drugalcdep.2007.11.015
36. Grothues, J. M., Bischof, G., Reinhardt, S., Meyer, C., John, U., & Rumpf, H.-J. (2008). Differences in help seeking rates after brief intervention for alcohol use disorders in general practice patients with and without comorbid anxiety or depressive disorders. *International Journal of Methods in Psychiatric Research*, *17*(S1), S74–S77. https://doi.org/10.1002/mpr.253
37. Gryczynski, J., Carswell, S. B., O’Grady, K. E., Mitchell, S. G., & Schwartz, R. P. (2018). Gender and ethnic differences in primary care patients’ response to computerized vs. In-person brief intervention for illicit drug misuse. *Journal of Substance Abuse Treatment*, *84*, 50–56. https://doi.org/10.1016/j.jsat.2017.10.009
38. Gryczynski, J., O’Grady, K. E., Mitchell, S. G., Ondersma, S. J., & Schwartz, R. P. (2016). Immediate versus delayed computerized brief intervention for illicit drug misuse. *Journal of Addiction Medicine*, *10*(5), 344–351. https://doi.org/10.1097/ADM.0000000000000248
39. Harris, S. K., Sherritt, L., Grubb, L., Samuels, R., Silva, T. J., Vernacchio, L., Wornham, W., Gibson, E., Levinson, J., & Knight, J. R. (2019). A randomized controlled trial of primary care screening and brief clinician intervention to reduce adolescents’ riding with an intoxicated driver. *Journal of Adolescent Health*, *64*(2), S7. https://doi.org/10.1016/j.jadohealth.2018.10.027
40. Harris, S. K., Sherritt, L., Grubb, L., Samuels, R., Silva, T., Vernacchio, L., Wornham, W., Erdem, G., & Knight, J. R. (2018). Practical tools to support adolescent substance abuse prevention in primary care: A multi-site randomized controlled trial of computer-facilitated screening and provider brief advice in the medical office. *Journal of Adolescent Health*, *62*(2), S13. https://doi.org/10.1016/j.jadohealth.2017.11.027
41. Heather, N., Bowie, A., Ashton, H., McAvoy, B., Spencer, I., Brodie, J., & Giddings, D. (2004). Randomised controlled trial of two brief interventions against long-term benzodiazepine use: Outcome of intervention. *Addiction Research & Theory*, *12*(2), 141–154. https://doi.org/10.1080/1606635310001634528
42. Horn, B. P., Crandall, C., Forcehimes, A., French, M. T., & Bogenschutz, M. (2017). Benefit-cost analysis of SBIRT interventions for substance using patients in emergency departments. *Journal of Substance Abuse Treatment*, *79*, 6–11. https://doi.org/10.1016/j.jsat.2017.05.003
43. Johnson, N. A., Kypri, K., Latter, J., McElduff, P., Attia, J., Saitz, R., Saunders, J. B., Wolfenden, L., Dunlop, A., Doran, C., & McCambridge, J. (2015). Effect of telephone follow-up on retention and balance in an alcohol intervention trial. *Preventive Medicine Reports*, *2*, 746–749. https://doi.org/10.1016/j.pmedr.2015.08.016
44. Johnson, N. A., Kypri, K., Saunders, J. B., Saitz, R., Attia, J., Dunlop, A., Doran, C., McElduff, P., Wolfenden, L., & McCambridge, J. (2013). The hospital outpatient alcohol project (HOAP): Protocol for an individually randomized, parallel-group superiority trial of electronic alcohol screening and brief intervention versus screening alone for unhealthy alcohol use. *Addiction Science & Clinical Practice*, *8*(1), 14. https://doi.org/10.1186/1940-0640-8-14
45. Johnson, N. A., Kypri, K., Saunders, J. B., Saitz, R., Attia, J., Latter, J., McElduff, P., Dunlop, A., Doran, C., Wolfenden, L., & McCambridge, J. (2018a). Effect of electronic screening and brief intervention on hazardous or harmful drinking among adults in the hospital outpatient setting: A randomized, double-blind, controlled trial. *Drug and Alcohol Dependence*, *191*, 78–85. https://doi.org/10.1016/j.drugalcdep.2018.06.030
46. Johnson, N. A., Kypri, K., Saunders, J. B., Saitz, R., Attia, J., Latter, J., McElduff, P., Dunlop, A., Doran, C., Wolfenden, L., & McCambridge, J. (2018b). Effect of electronic screening and brief intervention on hazardous or harmful drinking among adults in the hospital outpatient setting: A randomized, double-blind, controlled trial. *Drug and Alcohol Dependence*, *191*, 78–85. https://doi.org/10.1016/j.drugalcdep.2018.06.030
47. Kim, T. W., Bernstein, J., Cheng, D. M., Lloyd-Travaglini, C., Samet, J. H., Palfai, T. P., & Saitz, R. (2017). Receipt of addiction treatment as a consequence of a brief intervention for drug use in primary care: A randomized trial: Addiction treatment and brief intervention. *Addiction*, *112*(5), 818–827. https://doi.org/10.1111/add.13701
48. Knight, J. R., Sherritt, L., Gibson, E. B., Levinson, J. A., Grubb, L. K., Samuels, R. C., Silva, T., Vernacchio, L., Wornham, W., & Harris, S. K. (2019). Effect of computer-based substance use screening and brief behavioral counseling vs usual aare for youths in pediatric primary care: A pilot randomized clinical trial. *JAMA Network Open*, *2*(6), e196258. https://doi.org/10.1001/jamanetworkopen.2019.6258
49. Korcha, R. A., Cherpitel, C. J., Moskalewicz, J., Swiatkiewicz, G., Bond, J., & Ye, Y. (2012). Readiness to change, drinking, and negative consequences among Polish SBIRT patients. *Addictive Behaviors*, *37*(3), 287–292. https://doi.org/10.1016/j.addbeh.2011.11.006
50. Laporte, C., Lambert, C., Pereira, B., Blanc, O., Authier, N., Balayssac, D., Brousse, G., & Vorilhon, P. (2019). Cannabis users: Screen systematically, treat individually. A descriptive study of participants in a randomized trial in primary care. *PLOS ONE*, *14*(12), e0224867. https://doi.org/10.1371/journal.pone.0224867
51. Laporte, C., Vaillant-Roussel, H., Pereira, B., Blanc, O., Eschalier, B., Kinouani, S., Brousse, G., Llorca, P.-M., & Vorilhon, P. (2017). Cannabis and young esers—A brief intervention to reduce their consumption (CANABIC): A cluster randomized controlled trial in primary care. *The Annals of Family Medicine*, *15*(2), 131–139. https://doi.org/10.1370/afm.2003
52. Laporte, C., Vaillant-Roussel, H., Pereira, B., Blanc, O., Tanguy, G., Frappé, P., Costa, D., Gaboreau, Y., Badin, M., Marty, L., Clément, G., Dubray, C., Falissard, B., Llorca, P.-M., & Vorilhon, P. (2014). CANABIC: Cannabis and adolescents: Effect of a brief intervention on their consumption – study protocol for a randomized controlled trial. *Trials*, *15*(1), 40. https://doi.org/10.1186/1745-6215-15-40
53. Mason, M. J., Sabo, R., & Zaharakis, N. M. (2017). Peer network counseling as brief treatment for urban adolescent heavy cannabis users. *Journal of Studies on Alcohol and Drugs*, *78*(1), 152–157. https://doi.org/10.15288/jsad.2017.78.152
54. Mason, M., Light, J., Campbell, L., Keyser-Marcus, L., Crewe, S., Way, T., Saunders, H., King, L., Zaharakis, N. M., & McHenry, C. (2015). Peer network counseling with urban adolescents: A randomized controlled trial with moderate substance users. *Journal of Substance Abuse Treatment*, *58*, 16–24. https://doi.org/10.1016/j.jsat.2015.06.013
55. Maynié-François, C., Cheng, D. M., Samet, J. H., Lloyd-Travaglini, C., Palfai, T., Bernstein, J., & Saitz, R. (2017). Unhealthy alcohol use in primary care patients who screen positive for drug use. *Substance Abuse*, *38*(3), 303–308. https://doi.org/10.1080/08897077.2016.1216920
56. Meli, S., Palfai, T., Cheng, D. M., Alford, D., Bernstein, J., Samet, J., & Saitz, R. (2015). Screening and brief intervention for low risk drug use in primary care: A pilot randomized trial. *Drug and Alcohol Dependence*, *156*, e149–e150. https://doi.org/10.1016/j.drugalcdep.2015.07.407
57. Myers, B., van der Westhuizen, C., Naledi, T., Stein, D. J., & Sorsdahl, K. (2016). Readiness to change is a predictor of reduced substance use involvement: Findings from a randomized controlled trial of patients attending South African emergency departments. *BMC Psychiatry*, *16*(1), 35. https://doi.org/10.1186/s12888-016-0742-8
58. Nayak, M. B., Bond, J. C., Ye, Y., Cherpitel, C. J., Woolard, R., Bernstein, E., Bernstein, J., Villalobos, S., & Ramos, R. (2015). Readiness to change and to accept help and drinking outcomes in young adults of Mexican origin. *Journal of Studies on Alcohol and Drugs*, *76*(4), 602–606. https://doi.org/10.15288/jsad.2015.76.602
59. Neighbors, C. J., Colby, S. M., & Monti, P. M. (2010). Cost-Effectiveness of a Motivational Intervention for Alcohol-Involved Youth in a Hospital Emergency Department. *JOURNAL OF STUDIES ON ALCOHOL AND DRUGS*, 11.
60. Otto, C., Crackau, B., Löhrmann, I., Zahradnik, A., Bischof, G., John, U., & Rumpf, H.-J. (2009). Brief intervention in general hospital for problematic prescription drug use: 12-Month outcome. *Drug and Alcohol Dependence*, *105*(3), 221–226. https://doi.org/10.1016/j.drugalcdep.2009.07.010
61. Oviedo Ramirez, S., Alvarez, M. J., Field, C., Morera, O. F., Cherpitel, C., & Woolard, R. (2018). Brief intervention among Mexican-origin young adults in the emergency department at the USA–Mexico border: Examining the eole of patient’s preferred language of intervention in predicting drinking outcomes. *Alcohol and Alcoholism*, *53*(6), 728–734. https://doi.org/10.1093/alcalc/agy060
62. Palfai, T. P., Cheng, D. M., Bernstein, J. A., Palmisano, J., Lloyd-Travaglini, C. A., Goodness, T., & Saitz, R. (2016). Is the quality of brief motivational interventions for drug use in primary care associated with subsequent drug use? *Addictive Behaviors*, *56*, 8–14. https://doi.org/10.1016/j.addbeh.2015.12.018
63. Palfi, T., Cheng, D., Samet, J., Kraemer, K., Roberts, M., & Saitz, R. (2007). Depressive symptoms and subsequent alcohol use and problems: A prospective study of medical inpatients with unhealthy alcohol use. *Journal of Studies on Alcohol and Drugs*, *68*.
64. Park, T. W., Cheng, D. M., Lloyd-Travaglini, C. A., Bernstein, J., Palfai, T. P., & Saitz, R. (2015). Changes in health outcomes as a function of abstinence and reduction in illicit psychoactive drug use: A prospective study in primary care: Drug use and health outcomes in primary care. *Addiction*, *110*(9), 1476–1483. https://doi.org/10.1111/add.13020
65. Reddy, A., Vahidi, M., Cox, N., Alden, H., Andersen, R., & Gelberg, L. (2015). Evaluating the documentation of risky substance use in federally qualified health centers. *Drug and Alcohol Dependence*, *156*, e187. https://doi.org/10.1016/j.drugalcdep.2015.07.503
66. Reinhardt, S., G., Grothues, J., John, U., Meyer, C., & Rumpf, H. -j. (2008). Gender differences in the efficacy of brief interventions with a stepped care approach in general practice patients with alcohol-related disorders. *Alcohol and Alcoholism*, *43*(3), 334–340. https://doi.org/10.1093/alcalc/agn004
67. Rhodes, K. V., Rodgers, M., Sommers, M., Hanlon, A., Chittams, J., Doyle, A., Datner, E., & Crits-Christoph, P. (2015). Brief motivational intervention for intimate partner violence and heavy drinking in the emergency department: A randomized clinical trial. *JAMA*, *314*(5), 466. https://doi.org/10.1001/jama.2015.8369
68. Rhodes, K. V., Rodgers, M., Sommers, M., Hanlon, A., & Crits-Christoph, P. (2014). The Social Health Intervention Project (SHIP): Protocol for a randomized controlled clinical trial assessing the effectiveness of a brief motivational intervention for problem drinking and intimate partner violence in an urban emergency department. *BMC Emergency Medicine*, *14*(1), 10. https://doi.org/10.1186/1471-227X-14-10
69. Rose, G. L., Badger, G. J., Skelly, J. M., Ferraro, T. A., MacLean, C. D., & Helzer, J. E. (2016). A randomized controlled trial of IVR-based alcohol brief intervention to promote patient–provider communication in primary care. *Journal of General Internal Medicine*, *31*(9), 996–1003. https://doi.org/10.1007/s11606-016-3692-4
70. Saitz, R., Horton, N. J., Sullivan, L. M., Moskowitz, M. A., & Samet, J. H. (2003). Addressing alcohol problems in primary Care: A cluster randomized, controlled trial of a systems intervention: The Screening and Intervention in Primary Care (SIP) Study. *Annals of Internal Medicine*, *138*(5), 372. https://doi.org/10.7326/0003-4819-138-5-200303040-00006
71. Saitz, R., Kim, T., Bernstein, J., Cheng, D. M., Samet, J., Lloyd-Travaglini, C., Palfai, T., & German, J. (2015). Does screening and brief intervention for drug use in primary care increase receipt of substance use disorder treatment? *Addiction Science & Clinical Practice*, *10*(S2), O46. https://doi.org/10.1186/1940-0640-10-S2-O46
72. Saitz, R., Palfai, T. P. A., Cheng, D. M., Alford, D. P., Bernstein, J. A., Lloyd-Travaglini, C. A., Meli, S. M., Chaisson, C. E., & Samet, J. H. (2014). Screening and Brief Intervention for Drug Use in Primary Care: The ASPIRE Randomized Clinical Trial. *JAMA*, *312*(5), 502. https://doi.org/10.1001/jama.2014.7862
73. Saitz, R., Palfai, T. P., Cheng, D. M., Horton, N. J., Dukes, K., Kraemer, K. L., Roberts, M. S., Guerriero, R. T., & Samet, J. H. (2009). Some medical inpatients with unhealthy alcohol use may benefit from brief intervention. *Journal of Studies on Alcohol and Drugs*, *70*(3), 426–435. https://doi.org/10.15288/jsad.2009.70.426
74. Sorsdahl, K., Stein, D. J., Corrigall, J., Cuijpers, P., Smits, N., Naledi, T., & Myers, B. (2015). The efficacy of a blended motivational interviewing and problem solving therapy intervention to reduce substance use among patients presenting for emergency services in South Africa: A randomized controlled trial. *Substance Abuse Treatment, Prevention, and Policy*, *10*(1), 46. https://doi.org/10.1186/s13011-015-0042-1
75. Spirito, A., Monti, P. M., Barnett, N. P., Colby, S. M., Sindelar, H., Rohsenow, D. J., Lewander, W., & Myers, M. (2004). A randomized clinical trial of a brief motivational intervention for alcohol-positive adolescents treated in an emergency department. *The Journal of Pediatrics*, *145*(3), 396–402. https://doi.org/10.1016/j.jpeds.2004.04.057
76. Stern, S. A., Meredith, L. S., Gholson, J., Gore, P., & D’Amico, E. J. (2007). Project CHAT: A brief motivational substance abuse intervention for teens in primary care. *Journal of Substance Abuse Treatment*, *32*(2), 153–165. https://doi.org/10.1016/j.jsat.2006.07.009
77. Vahidi, M., Rico, M., Scholtz, J., Garcia, M., Andersen, R., Yacenda, J., & Gelberg, L. (2015). Feasibility of the quit using drugs intervention trial. *Drug and Alcohol Dependence*, *146*, e8. https://doi.org/10.1016/j.drugalcdep.2014.09.704
78. Walter, A. W., Cheng, D. M., Lloyd-Travaglini, C. A., Samet, J. H., Bernstein, J., & Saitz, R. (2016). Are decreases in drug use risk associated with reductions in HIV sex risk behaviors among adults in an urban hospital primary care setting? *Preventive Medicine Reports*, *4*, 410–416. https://doi.org/10.1016/j.pmedr.2016.08.001
79. Zahradnik, A., Otto, C., Crackau, B., Löhrmann, I., Bischof, G., John, U., & Rumpf, H.-J. (2009). Randomized controlled trial of a brief intervention for problematic prescription drug use in non-treatment-seeking patients. *Addiction*, *104*(1), 109–117. https://doi.org/10.1111/j.1360-0443.2008.02421.x
80. Zarkin, G., Bray, J., Hinde, J., & Saitz, R. (2015). Costs of screening and brief intervention for illicit drug use in primary care settings. *Journal of Studies on Alcohol and Drugs*, *76*(2), 222–228. https://doi.org/10.15288/jsad.2015.76.222
