# Supplementary material for: Investigating for Whom Brief Substance Use Interventions Are Most Effective: An Individual Participant Data Meta-analysis
Source: Prev Sci. 2023 May 3;24(8):1459–82. doi: 10.1007/s11121-023-01525-1 (PMC10678844; doi:10.1007/s11121-023-01525-1)
Supplement: Supplementary file 6 — Supplementary file6 (DOCX 27 KB) [file 11121_2023_1525_MOESM6_ESM.docx]

| **Supplemental File 6 – Included Study Characteristics** | | | | | | | | | | | | | |
| --- | --- | --- | --- | --- | --- | --- | --- | --- | --- | --- | --- | --- | --- |
| **Study Name; Primary Author** | **Study Characteristics** | | | | **Sample Characteristics** | | | **Intervention Characteristics** | | | | | |
|  | Country | Sample Type | Design | Follow-up (in months)^a^ | Mean Age  (range) | Race/Ethnicity  Category - %^b^ | Gender (% female) | Setting^b^ | Modality | Booster (#)^c^ | Duration (minutes)^c^ | Provider Type | Int. target |
| Project STRIVE, Sorsdahl | South Africa | Screened/Risk Sample | RCT | 3 | 28 (18-75) | Black – 58%; Nonwhite – 41%; White – 1% | 35% | Emergency department | In-person | 0 | 20 | Peer counselor or educator | Mixed |
| Project CHAT (2008), D’Amico | U.S. | Screened/Risk Sample | RCT | 3,6,12 | 15 (12-18) | Latinx – 85.7%; Black – 9.5%; White – 4.8% | 52% | Community Health | In-person | 1 | 15-20 | Behavior Specialist | Mixed |
| Project CHAT, D’Amico | U.S. | Screened/Risk Sample | RCT | 3 | 16 (12-18) | Nonwhite – 71% Black – 17%; White - 12% | 52% | Community Health | In-person | 1 | 15-20 | Behavior Specialist | Mixed |
| HaLT-Hamburg, Arnaud | Germany | Screened/Risk Sample | RCT | 3,6 | 15.76 (12-17) | NR | 49% | Community Health | In-person | 1 | 45 | Behavior Specialist | Alcohol |
| Harris | U.S. | Universal/Unscreened sample | RCT | 3,6,12 | 16.4 (12-18) | Nonwhite – 50%; White – 50% | 54% | Community Health, Outpatient | In-person | 0 | 7.5 | Primary Care Provider | Mixed |
| Daeppen | Switzerland | Screened/Risk Sample | RCT | 12 | 36.7 (18- ) | NR | 24% | Emergency department | In-person | 0 | 17.1 | Behavior Specialist | Alcohol |
| Barticevic | Chile | Screened/Risk Sample | RCT | 6 | 29 (18-45) | NR | 43% | Community Health | In-person | 0 | 5 | Other | Alcohol |
| SMART-ED, Bogneschutz | U.S. | Screened/Risk Sample | RCT | 3,6,12 | 36 (18-72) | Black -34%; Asian – 2%, Latinx – 24%, White – 5% | 30% | Emergency department | In-person | 2 | 30 | Other | Mixed |
| ASPIRE, Saitz | U.S. | Screened/Risk Sample | RCT | 6 | 41.3 (18 - ) | Black – 69%; White – 20%; Latinx – 11% | 30% | University, Outpatient | In-person | 0; 1* | 10-15; 30-45* | Other, Behavior Specialist* | Mixed |
| Rose | U.S. | Screened/Risk Sample | RCT | 3,6 | 46.9 (18-87) | Nonwhite – 5%; White – 95% | 52% | University, Outpatient | Telephone | 0 | 6.5 | Other | Alcohol |
| Spirito | U.S. | Screened/Risk Sample | RCT | 3,6,12 | 15.6 (13-17) | Black – 7%; Asian – 4%; Latinx – 17%; White – 72% | 36% | Emergency department | In-person | 0 | 35-45 | Behavior Specialist | Alcohol |
| Heather | England | Universal/Unscreened Sample | RCT | 6 | 69.16 (40-96) | NR | 77% | Private provider, Other | Other, In-person* | 0 | 2, 12* | Primary Care Provider | Drug |
| Project QUIT, Gelberg | U.S. | Screened/Risk Sample | RCT | 3 | 41.7 (18 - ) | Other– 23%; White – 30%; Black – 47%; | 37% | Community Health | In-person | 2 | 3-4 | Primary Care Provider | Mixed |
| QUIT replication, Gelberg | U.S. | Screened/Risk Sample | RCT | 3 | 30.8 (18 - ) | Latinx – 93%  Not Hispanic – 7% | 41% | Community Health | In-person | 2 | 3-4 | Primary Care Provider | Drugs |
| Peer Network Counseling, Mason | U.S. | Screened/Risk Sample | RCT | 3,6 | 16.4 (14-18) | Black – 84%; Other – 16% | 71% | Community Health, University | In-person | 0 | 20 | Behavior Specialist | Mixed |
| Gryczynski | U.S. | Screened/Risk Sample | RCT | 3 | 35 (18-62) | NR | 53% | Community Health | Laptop/  computer/tablet | 0 | 8.7 | Other provider | Drugs |
| SIP, Bischof | Germany | Screened/Risk Sample | RCT | 12 | 36.49 (NR) | NR | 32% | Community Health | Telephone | 0 | 30-160, 120* | Behavior Specialist | Alcohol |
| HOAP, Johnson | Australia | Screened/Risk Sample | RCT | 6,12 | 44.1 (18-89) | NR | 25% | Outpatient | Laptop/  computer/tablet | 0 | 5-10 | Other provider | Alcohol |
| Cherpitel (2016) | U.S. | Screened/Risk Sample | RCT | 3.12 | 23.96 (18-30) | Latinx – 100% | 44% | Emergency department | In-person | 0 | 20 | Peer | Alcohol |
| Cherpitel | Poland | Screened/Risk Sample | RCT | 3,12 | 35.05 (18-77) | NR | 15% | Emergency department | In-person | 0 | 15-20 | General Practitioner | Alcohol |
| Assanangkornchai | Thailand | Screened/Risk Sample | RCT | 3,6 | 40.5 (16-65) | Asian – 100% | 20% | Community Health, Outpatient | In-person | 0 | 8.8 | General Practitioner | Mixed |
| Ponce | Argentina | Screened/Risk Sample | RCT | 3 | 38.8 (18-78) | Asian – 5%; White – 35%; Nonwhite – 30%; Black – 30% | 100% | Community | In-person | 0 | 20-30 | Behavior Specialist, Graduate Student | Mixed |
| SIP, Saitz | U.S. | Screened/Risk Sample | RCT | 6 | 43.01 (18 -) | Black – 56%; White – 19%; Latinx – 17% | 37% | University | In-person | 0 | NR | Primary Care Provider | Alcohol |
| ASAP, Saitz | U.S. | Screened/Risk Sample | RCT | 3,12 | 44 (18 - ) | Black – 46.3%; White – 37.8%; Latinx – 9% | 39% | Inpatient Setting | In-person | 0 | 30 | Graduate Student/Trainee | Alcohol |
| Rhoades | U.S. | Screened/Risk Sample | RCT | 3,6,12 | 32.1 (18-64) | Black – 80.1%; White – 18.4%; Native America – 3.4%; Hispanic – 4.8%; Asian – 1.9%; Other – 6%; Missing – 0.7% | 100% | Emergency department | In-person | 1 | 20-30 | Behavior Specialist | Mixed |
| Monti | U.S. | Screened/Risk Sample | RCT | 3,6 | 18.4 (18-19) | Latinx – 2%; Asian – 5%; Black – 13%; White = 80% | 37% | Emergency department | In-person | 0 | 35-40 | Behavior Specialist | Alcohol |
| CANABIC, Laporte | France | Screened/Risk Sample | RCT | 3,6,12 | 20.6 (15-25) | NR | 35% | Community Health | In-person | 0 | NR | Primary Care Provider | Drugs |
| Bruguera | Spain | Screened/Risk Sample | RCT | 3 | 43 (NR) | NR | 28% | Emergency department | In-person | 0 | 5-15 | Behavior Specialist | Alcohol |
| MIMIK, Zahradnik | Germany | Screened/Risk Sample | RCT | 3,12 | 55.13 (30-62) | NR | 62% | Community health, University, Inpatient | In-person | 1 | 30-45 | Behavior Specialist | Mixed |
| *Note.* NR = not reported; Int = Intervention  ^a^ Timeframes listed were those used in this study  ^b^ Categories were coded and collapsed according to available data and do not necessarily represent those provided by original authors.  ^c^ Where indicated by an *, two interventions conditions were compared and therefore intervention length varied accordingly. | | | | | | | | | | | | | |
