# Supplementary material for: Investigating for Whom Brief Substance Use Interventions Are Most Effective: An Individual Participant Data Meta-analysis
Source: Prev Sci. 2023 May 3;24(8):1459–82. doi: 10.1007/s11121-023-01525-1 (PMC10678844; doi:10.1007/s11121-023-01525-1)
Supplement: Supplementary file 7 — Supplementary file7 (DOCX 140 KB) [file 11121_2023_1525_MOESM7_ESM.docx]

|  |  |  |  |  |  |  |  |  |  |  |  |  |  |  |
| --- | --- | --- | --- | --- | --- | --- | --- | --- | --- | --- | --- | --- | --- | --- |
| **Supplemental Table 1** | | | | | | | | | | | | | | |
| Pooled pretest-adjusted standardized effect sizes, 95% confidence intervals, and heterogeneity statistics by each moderator subgroup, for Binge Alcohol Consumption at 3, 6, and 9-12-months post-baseline | | | | | | | | | | | | | | |
|  | | Follow-up Time | | | | | | | | | | | | |
|  | | 3 month | | |  | 6 month | | | |  | | 9-12 months | | |
|  | | $\bar{ES}$ [95% CI] | $\tau^{2}$ | $I^{2}$ (%) |  | $\bar{ES}$ [95% CI] | $\tau^{2}$ | $I^{2}$ (%) |  | | $\bar{ES}$ [95% CI] | | $\tau^{2}$ | $I^{2}$ (%) |
| **Age** | |  |  |  |  |  |  |  |  | |  | |  |  |
| Adolescent | | 0.00 [-0.22, 0.23]_3_ | 0.00 | 0.00 |  | — | — | — |  | | — | | — | — |
| Young adult | | 0.02 [-0.13, 0.16]_8_ | 0.00 | 0.00 |  | -0.01 [-0.13, 0.10]^­­­­^­­­_6_ | 0.00 | 0.00 |  | | 0.01 [-0.20, 0.21]_6_ | | 0.00 | 0.00 |
| Adult | | 0.06 [-0.01, 0.13]_5_ | 0.00 | 0.00 |  | -0.00 [-0.09, 0.10]_4_ | 0.00 | 0.00 |  | | 0.02 [-0.11, 0.15]_5_ | | 0.00 | 0.00 |
| **Sex** | |  |  |  |  |  |  |  |  | |  | |  |  |
| Female | | **0.09 [0.03, 0.14]_10,*_** | 0.00 | 0.00 |  | 0.01 [-0.05, 0.08]_8_ | 0.00 | 0.00 |  | | 0.07 [-0.05, 0.20]_7_ | | 0.00 | 0.00 |
| Male | | 0.03 [-0.04, 0.10]_10_ | 0.00 | 0.00 |  | -0.01 [-0.05, 0.04]_8_ | 0.00 | 0.00 |  | | -0.04 [-0.20, 0.12]_7_ | | 0.00 | 0.00 |
| **Employment** | |  |  |  |  |  |  |  |  | |  | |  |  |
| Unemployed | | **-0.07 [-0.13, 0.00]_3_** | 0.00 | 0.00 |  | 0.01 [-0.78, 0.79]_2_ | 0.00 | 0.00 |  | | -0.06 [-0.29, 0.17]_3_ | | 0.00 | 0.00 |
| Employed | | -0.01 [-0.23, 0.22]_3_ | 0.00 | 0.00 |  | **-0.09 [-0.11, -0.07]_2_** | 0.00 | 0.00 |  | | 0.03 [-0.16, 0.22]_3_ | | 0.00 | 0.00 |
| **Relationship Status** | |  |  |  |  |  |  |  |  | |  | |  |  |
| Single | | 0.02 [-0.09, 0.14]_4_ | 0.00 | 0.00 |  | 0.00 [-0.04, 0.04]_4_ | 0.00 | 0.00 |  | | -0.03 [-0.19, 0.12]_3_ | | 0.00 | 0.00 |
| Married | | 0.09 [0.00, 0.18]_4_ | 0.00 | 0.00 |  | -0.05 [-0.29, 0.20]_4_ | 0.02 | 31.25 |  | | 0.15 [-0.45, 0.74]_3_ | | 0.00 | 0.00 |
| **Education** | |  |  |  |  |  |  |  |  | |  | |  |  |
| Below High School | | 0.11 [-0.05, 0.27]_5_ | 0.00 | 0.00 |  | -0.01 [-0.13, 0.12]_6_ | 0.00 | 0.00 |  | | 0.19 [-0.22, 0.60]_4_ | | 0.00 | 0.00 |
| High School  Diploma or above | | 0.04 [-0.05, 0.14]_5_ | 0.00 | 0.00 |  | 0.01 [-0.16, 0.18]_5_ | 0.00 | 4.34 |  | | -0.03 [-0.25, 0.18]_4_ | | 0.00 | 0.00 |
| **BL Severity Substance Use** | | |  |  |  |  |  |  |  | |  | |  |  |
| Low severity | | -0.03 [-0.18, 0.12]_3_ | 0.00 | 0.00 |  | -0.03 [-0.33, 0.27]_3_ | 0.00 | 0.00 |  | | -0.03 [-0.23, 0.17]_2_ | | 0.00 | 0.00 |
| Moderate severity | | 0.33 [-0.65, 1.31]_3_ | 0.05 | 26.27 |  | -0.16 [-0.76, 0.43]_3_ | 0.02 | 29.37 |  | | -0.21 [-1.93, 1.51]_2_ | | 0.00 | 0.00 |
| High severity | | -0.58 [-2.55, 1.38]_2_ | 0.00 | 0.00 |  | -0.22 [-0.54, 0.11]_2_ | 0.00 | 0.00 |  | | — | | — | — |
| *Note*. Results in boldface are significantly different from null value (i.e., indicating no marginal mean difference between intervention and comparison conditions within a specific moderator subgroup). * subscript indicates significant values after Benjamini-Hochberg adjustment for multiple comparisons. $\bar{ES}$ = average effect size. CI = 95% confidence interval with robust standard errors. — indicates results not available due to inadequate effect sizes. Subscripts indicate *k* (number of studies). | | | | | | | | | | | | | | |

| **Supplemental Table 2** | | | | | | | | | | | | | |
| --- | --- | --- | --- | --- | --- | --- | --- | --- | --- | --- | --- | --- | --- |
| Pooled pretest-adjusted standardized effect sizes, 95% confidence intervals, and heterogeneity statistics by each moderator subgroup, for frequency of alcohol consumption at 3, 6, and 9-12-months post-baseline | | | | | | | | | | | | | |
|  | Follow-up Time | | | | | | | | | | | | |
|  | 3 month | | |  | 6 month | | | |  | | 9-12 months | | |
|  | $\bar{ES}$ [95% CI] | $\tau^{2}$ | $I^{2}$ (%) |  | $\bar{ES}$ [95% CI] | $\tau^{2}$ | $I^{2}$ (%) |  | | $\bar{ES}$ [95% CI] | | $\tau^{2}$ | $I^{2}$ (%) |
| **Age** |  |  |  |  |  |  |  |  | |  | |  |  |
| Adolescent | 0.09 [-0.08, 0.26]_3_ | 0.00 | 0.00 |  | 0.22 [-0.07, 0.51]_2_ | 0.00 | 0.00 |  | | — | | — | — |
| Young adult | 0.07 [-0.06, 0.20]_9_ | 0.00 | 0.00 |  | 0.07 [-0.19, 0.34]_4_ | 0.00 | 0.00 |  | | -0.01 [-0.51, 0.48]_4_ | | 0.01 | 17.76 |
| Adult | 0.01 [-0.05, 0.07]_6_ | 0.00 | 0.00 |  | 0.04 [-0.38,0.47]_2_ | 0.00 | 0.00 |  | | 0.01 [-0.52, 0.54]_2_ | | 0.03 | 65.60 |
| **Sex** |  |  |  |  |  |  |  |  | |  | |  |  |
| Female | **0.10 [0.03, 0.17]_11, *_** | 0.00 | 0.00 |  | **0.08 [0.00, 0.15]_6_** | 0.00 | 0.00 |  | | 0.07 [-0.17, 0.30]_6_ | | 0.02 | 46.98 |
| Male | 0.02 [-0.04, 0.08]_11_ | 0.00 | 0.00 |  | 0.03 [-0.10, 0.16]_6_ | 0.00 | 0.03 |  | | 0.00 [-0.20, 0.20]_6_ | | 0.01 | 21.02 |
| **Employment** |  |  |  |  |  |  |  |  | |  | |  |  |
| Unemployed | 0.03 [-0.18, 0.25]_4_ | 0.00 | 0.00 |  | — | — | — |  | | — | | — | — |
| Employed | 0.00 [-0.09, 0.10]_4_ | 0.00 | 0.00 |  | — | — | — |  | | — | | — | — |
| **Relationship Status** |  |  |  |  |  |  |  |  | |  | |  |  |
| Single | 0.01 [-0.04, 0.06]_4_ | 0.00 | 0.00 |  | **0.06 [0.03, 0.08]_2_** | 0.00 | 0.00 |  | | -0.05 [-1.42, 1.32]_2_ | | 0.00 | 0.00 |
| Married | 0.06 [-0.04, 0.16]_4_ | 0.00 | 0.00 |  | 0.00 [-1.80, 1.80]_2_ | 0.02 | 30.11 |  | | 0.35 [-0.89, 1.60]_2_ | | 0.00 | 0.00 |
| **Education** |  |  |  |  |  |  |  |  | |  | |  |  |
| Below High School | **0.16 [0.09, 0.22]_6, *_** | 0.00 | 0.00 |  | 0.17 [-0.11, 0.44]_4_ | 0.00 | 3.72 |  | | **0.17 [0.04, 0.31]_3_** | | 0.00 | 0.00 |
| High School  Diploma or above | 0.01 [-0.11, 0.13]_6_ | 0.00 | 0.00 |  | 0.37 [-4.38, 5.13]_3_ | 2.71 | 88.34 |  | | 0.68 [-3.14, 4.51]_3_ | | 1.57 | 97.42 |
| **BL Severity Substance Use** | |  |  |  |  |  |  |  | |  | |  |  |
| Low severity | 0.07 [-0.04, 0.18]_3_ | 0.00 | 0.00 |  | — | — | — |  | | — | | — | — |
| Moderate severity | 0.31 [-0.81, 1.43]_3_ | 0.12 | 50.56 |  | — | — | — |  | | — | | — | — |
| High severity | 0.07 [-1.65, 1.79]_2_ | 0.00 | 0.00 |  | — | — | — |  | | — | | — | — |
| *Note*. Results in boldface are significantly different from null value (i.e., indicating no marginal mean difference between intervention and comparison conditions within a specific moderator subgroup). * subscript indicates significant values after Benjamini-Hochberg adjustment for multiple comparisons. $\bar{ES}$ = average effect size. CI = 95% confidence interval with robust standard errors. — indicates results not available due to inadequate effect sizes. Subscripts indicate *k* (number of studies). | | | | | | | | | | | | | |

| **Supplemental Table 3** | | | | | | | | | | | | | |
| --- | --- | --- | --- | --- | --- | --- | --- | --- | --- | --- | --- | --- | --- |
| Pooled pretest-adjusted standardized effect sizes, 95% confidence intervals, and heterogeneity statistics by each moderator subgroup, for quantity of alcohol consumption at 3, 6, and 9-12-months post-baseline | | | | | | | | | | | | | |
|  | Follow-up Time | | | | | | | | | | | | |
|  | 3 month | | |  | 6 month | | | |  | | 9-12 months | | |
|  | $\bar{ES}$ [95% CI] | $\tau^{2}$ | $I^{2}$ (%) |  | $\bar{ES}$ [95% CI] | $\tau^{2}$ | $I^{2}$ (%) |  | | $\bar{ES}$ [95% CI] | | $\tau^{2}$ | $I^{2}$ (%) |
| **Age** |  |  |  |  |  |  |  |  | |  | |  |  |
| Adolescent | — | — | — |  | — | — | — |  | | — | | — | — |
| Young adult | -0.01 [-0.08, 0.06]_10_ | 0.00 | 0.00 |  | -0.07 [-0.33, 0.18]_5_ | 0.01 | 22.43 |  | | -0.04 [-0.19, 0.11]_7_ | | 0.00 | 0.00 |
| Adult | 0.06 [-0.03, 0.14]_9_ | 0.00 | 0.00 |  | -0.01 [-0.09, 0.07]_5_ | 0.00 | 0.00 |  | | 0.00 [-0.06, 0.06]_7_ | | 0.00 | 0.00 |
| **Sex** |  |  |  |  |  |  |  |  | |  | |  |  |
| Female | 0.08 [-0.02, 0.18]_11_ | 0.00 | 0.00 |  | 0.00 [-0.24, 0.23]_5_ | 0.01 | 28.61 |  | | -0.05 [-0.16, 0.06]_7_ | | 0.00 | 0.00 |
| Male | 0.05 [-0.02, 0.11]_11_ | 0.00 | 0.00 |  | -0.07 [-0.24, 0.10]_5_ | 0.00 | 0.01 |  | | 0.02 [-0.10, 0.13]_7_ | | 0.00 | 0.00 |
| **Employment** |  |  |  |  |  |  |  |  | |  | |  |  |
| Unemployed | -0.02 [-0.22, 0.17]_5_ | 0.00 | 0.00 |  | -0.03 [-0.45, 0.38]_2_ | 0.00 | 0.00 |  | | -0.02 [-0.12, 0.09]_3_ | | 0.00 | 0.00 |
| Employed | 0.00 [-0.16, 0.16]_5_ | 0.00 | 0.00 |  | 0.11 [-1.18, 1.40]_2_ | 0.00 | 0.00 |  | | -0.02 [-0.15, 0.11]_3_ | | 0.00 | 0.00 |
| **Relationship Status** |  |  |  |  |  |  |  |  | |  | |  |  |
| Single | 0.03 [-0.18, 0.25]_5_ | 0.00 | 0.00 |  | -0.05 [-0.41, 0.32]_4_ | 0.04 | 70.84 |  | | 0.00 [-0.17, 0.16]_3_ | | 0.00 | 0.00 |
| Married | 0.04 [-0.05, 0.12]_5_ | 0.00 | 0.00 |  | -0.02 [-0.11, 0.08]_4_ | 0.00 | 0.00 |  | | 0.01 [-0.37, 0.39]_3_ | | 0.00 | 0.00 |
| **Education** |  |  |  |  |  |  |  |  | |  | |  |  |
| Below High School | 0.13 [-0.26, 0.51]_5_ | 0.04 | 36.67 |  | -0.05 [-0.28, 0.18]_4_ | 0.00 | 0.00 |  | | -0.06 [-0.62, 0.50]_3_ | | 0.00 | 0.00 |
| High School  Diploma or above | -0.01 [-0.23, 0.22]_5_ | 0.00 | 0.01 |  | -0.03 [-0.30, 0.23]_3_ | 0.00 | 7.65 |  | | 0.02 [-0.24, 0.28]_3_ | | 0.00 | 0.00 |
| **BL Severity Substance Use** | |  |  |  |  |  |  |  | |  | |  |  |
| Low severity | -0.02 [-0.54, 0.50]_3_ | 0.00 | 0.00 |  | — | — | — |  | | 0.06 [-0.37, 0.49]_2_ | | 0.00 | 0.00 |
| Moderate severity | -0.02 [-0.39, 0.35]_3_ | 0.00 | 0.00 |  | — | — | — |  | | -0.34 [-3.26, 2.58]_2_ | | 0.01 | 12.71 |
| High severity | -0.15 [-1.39, 1.08]_3_ | 0.12 | 38.28 |  | — | — | — |  | | — | | — | — |
| *Note*. $\bar{ES}$ = average effect size (marginal mean difference between intervention and comparison conditions within a specific moderator subgroup). CI = 95% confidence interval with robust standard errors. — indicates results not available due to inadequate effect sizes. Subscripts indicate *k* (number of studies). | | | | | | | | | | | | | |

| **Supplemental Table 4** | | | | | | | | | | | | | |
| --- | --- | --- | --- | --- | --- | --- | --- | --- | --- | --- | --- | --- | --- |
| Pooled pretest-adjusted standardized effect sizes, 95% confidence intervals, and heterogeneity statistics by each moderator subgroup, for frequency of cannabis consumption at 3, 6, and 9-12-months post-baseline | | | | | | | | | | | | | |
|  | Follow-up Time | | | | | | | | | | | | |
|  | 3 month | | |  | 6 month | | | |  | | 9-12 months | | |
|  | $\bar{ES}$ [95% CI] | $\tau^{2}$ | $I^{2}$ (%) |  | $\bar{ES}$ [95% CI] | $\tau^{2}$ | $I^{2}$ (%) |  | | $\bar{ES}$ [95% CI] | | $\tau^{2}$ | $I^{2}$ (%) |
| **Age** |  |  |  |  |  |  |  |  | |  | |  |  |
| Adolescent | 0.07 [ -0.18, 0.33]_3_ | 0.00 | 0.00 |  | -0.05 [-0.21, 0.11]_2_ | 0.00 | 0.00 |  | | — | | — | — |
| Young adult | 0.18 [-.015, 0.50]_5_ | 0.00 | 0.00 |  | -0.19 [-0.99, 0.60]_2_ | 0.00 | 0.00 |  | | — | | — | — |
| Adult | -0.02 [-1.63, 1.59]_2_ | 0.00 | 0.00 |  | — | — | — |  | | — | | — | — |
| **Sex** |  |  |  |  |  |  |  |  | |  | |  |  |
| Female | 0.03 [-0.14, 0.19]_6_ | 0.00 | 0.00 |  | -0.08 [-0.40, 0.24]_3_ | 0.00 | 0.00 |  | | -0.15 [-2.63, 2.33]_2_ | | 0.06 | 72.37 |
| Male | 0.00 [-0.21, 0.21]_6_ | 0.00 | 0.00 |  | -0.05 [-0.40, 0.30]_3_ | 0.00 | 0.00 |  | | -0.02 [-0.77, 0.73]_2_ | | 0.00 | 0.00 |
| **Education** |  |  |  |  |  |  |  |  | |  | |  |  |
| Below High School | 0.19 [-0.77, 1.15]_2_ | 0.00 | 0.00 |  | — | — | — | — | | — | | — | — |
| High School  Diploma or above | 0.21 [-5.72, 6.14]_2_ | 0.26 | 49.01 |  | — | — | — | — | | — | | — | — |
| **BL Severity Substance Use** | |  |  |  |  |  |  |  | |  | |  |  |
| Low severity | -0.04 [-0.12, 0.04]_3_ | 0.00 | 0.00 |  | — | — | — | — | | — | | — | — |
| Moderate severity | -0.19 [-0.80, 0.43]_3_ | 0.00 | 0.00 |  | — | — | — | — | | — | | — | — |
| High severity | 0.27 [-1.08, 1.63]_3_ | 0.00 | 0.00 |  | — | — | — | — | | — | | — | — |
| *Note*. $\bar{ES}$ = average effect size (marginal mean difference between intervention and comparison conditions within a specific moderator subgroup). CI = 95% confidence interval with robust standard errors. — indicates results not available due to inadequate effect sizes. Subscripts indicate *k* (number of studies). | | | | | | | | | | | | | |

| **Supplemental Table 5** | | | | | | | | | | | | | |
| --- | --- | --- | --- | --- | --- | --- | --- | --- | --- | --- | --- | --- | --- |
| Pooled pretest-adjusted standardized effect sizes, 95% confidence intervals, and heterogeneity statistics by each moderator subgroup, for quantity of cannabis consumption at 3, 6, and 9-12-months post-baseline | | | | | | | | | | | | | |
|  | Follow-up Time | | | | | | | | | | | | |
|  | 3 month | | |  | 6 month | | | |  | | 9-12 months | | |
|  | $\bar{ES}$ [95% CI] | $\tau^{2}$ | $I^{2}$ (%) |  | $\bar{ES}$ [95% CI] | $\tau^{2}$ | $I^{2}$ (%) |  | | $\bar{ES}$ [95% CI] | | $\tau^{2}$ | $I^{2}$ (%) |
| **Age** |  |  |  |  |  |  |  |  | |  | |  |  |
| Adolescent | -0.10 [-0.27, 0.06]_3_ | 0.00 | 0.00 |  | 0.15 [-0.05, 0.35]_2_ | 0.00 | 0.00 |  | | — | | — | — |
| Young adult | 0.38 [-0.75, 1.50]_4_ | 0.30 | 60.41 |  | -0.17 [-1.40, 1.06]_2_ | 0.00 | 0.00 |  | | — | | — | — |
| Adult | — | — | — |  | — | — | — |  | | — | | — | — |
| **Sex** |  |  |  |  |  |  |  |  | |  | |  |  |
| Female | 0.01 [-0.30, 0.31]_5_ | 0.00 | 0.00 |  | **0.11 [0.01, 0.21]_3_** | 0.00 | 0.00 |  | | -0.06 [-0.76, 0.64]_2_ | | 0.00 | 0.00 |
| Male | -0.04 [-0.21, 0.14]_5_ | 0.00 | 0.00 |  | -0.03 [-0.23, 0.16]_3_ | 0.00 | 0.00 |  | | -0.06 [-0.30, 0.19]_2_ | | 0.00 | 0.00 |
| *Note*. Results in boldface are significantly different from null value (i.e., indicating no marginal mean difference between intervention and comparison conditions within a specific moderator subgroup). * subscript indicates significant values after Benjamini-Hochberg adjustment for multiple comparisons. $\bar{ES}$ = average effect size. CI = 95% confidence interval with robust standard errors. — indicates results not available due to inadequate effect sizes. Subscripts indicate *k* (number of studies). | | | | | | | | | | | | | |

| **Supplemental Table 6** | | | | | | | | | | | | | |
| --- | --- | --- | --- | --- | --- | --- | --- | --- | --- | --- | --- | --- | --- |
| Pooled pretest-adjusted standardized effect sizes, 95% confidence intervals, and heterogeneity statistics by each moderator subgroup, for tobacco consumption at 3, 6, and 9-12-months post-baseline | | | | | | | | | | | | | |
|  | Follow-up Time | | | | | | | | | | | | |
|  | 3 month | | |  | 6 month | | | |  | | 9-12 months | | |
|  | $\bar{ES}$ [95% CI] | $\tau^{2}$ | $I^{2}$ (%) |  | $\bar{ES}$ [95% CI] | $\tau^{2}$ | $I^{2}$ (%) |  | | $\bar{ES}$ [95% CI] | | $\tau^{2}$ | $I^{2}$ (%) |
| **Age** |  |  |  |  |  |  |  |  | |  | |  |  |
| Adolescent | — | — | — |  | — | — | — |  | | — | | — | — |
| Young adult | 0.06 [-0.35, 0.47]_4_ | 0.00 | 0.00 |  | — | — | — |  | | — | | — | — |
| Adult | 0.07 [-0.14, 0.27]_3_ | 0.00 | 0.00 |  | — | — | — |  | | — | | — | — |
| **Sex** |  |  |  |  |  |  |  |  | |  | |  |  |
| Female | 0.08 [-0.06, 0.23]_6_ | 0.00 | 0.00 |  | -0.06 [-0.22, 0.09]_3_ | 0.00 | 0.00 |  | | -0.03 [-1.02, 0.95]_2_ | | 0.00 | 0.00 |
| Male | -0.02 [-0.20, 0.17]_6_ | 0.00 | 0.00 |  | 0.13 [-0.07, 0.32]_3_ | 0.00 | 0.00 |  | | 0.06 [-0.03, 0.16]_2_ | | 0.00 | 0.00 |
| **Education** |  |  |  |  |  |  |  |  | |  | |  |  |
| Below High School | 0.13 [-0.51, 0.77]_2_ | 0.00 | 0.00 |  | — | — | — |  | | — | | — | — |
| High School  Diploma or above | 0.52 [-7.99, 9.03]_2_ | 0.69 | 71.69 |  | — | — | — |  | | — | | — | — |
| **BL Severity Substance Use** | |  |  |  |  |  |  |  | |  | |  |  |
| Low severity | 0.00 [-0.05, 0.05]_3_ | 0.00 | 0.00 |  | — | — | — |  | | — | | — | — |
| Moderate severity | 0.27 [-0.37, 0.90]_3_ | 0.00 | 0.00 |  | — | — | — |  | | — | | — | — |
| High severity | -0.23 [-3.53, 3.07]_2_ | 0.00 | 0.00 |  | — | — | — |  | | — | | — | — |
| *Note*. $\bar{ES}$ = average effect size (marginal mean difference between intervention and comparison conditions within a specific moderator subgroup). CI = 95% confidence interval with robust standard errors. — indicates results not available due to inadequate effect sizes. Subscripts indicate *k* (number of studies). | | | | | | | | | | | | | |

| **Supplemental Table 7** | | | | | | | | | | | | | |
| --- | --- | --- | --- | --- | --- | --- | --- | --- | --- | --- | --- | --- | --- |
| Pooled pretest-adjusted standardized effect sizes, 95% confidence intervals, and heterogeneity statistics by each moderator subgroup, for other drug consumption at 3, 6, and 9-12-months post-baseline | | | | | | | | | | | | | |
|  | Follow-up Time | | | | | | | | | | | | |
|  | 3 month | | |  | 6 month | | | |  | | 9-12 months | | |
|  | $\bar{ES}$ [95% CI] | $\tau^{2}$ | $I^{2}$ (%) |  | $\bar{ES}$ [95% CI] | $\tau^{2}$ | $I^{2}$ (%) |  | | $\bar{ES}$ [95% CI] | | $\tau^{2}$ | $I^{2}$ (%) |
| **Age** |  |  |  |  |  |  |  |  | |  | |  |  |
| Adolescent | — | — | — |  | — | — | — |  | | — | | — | — |
| Young adult | -0.03 [-0.80, 0.73]_3_ | 0.00 | 0.00 |  | -0.50 [-1.58, 0.58]_2_ | 0.00 | 0.00 |  | | — | | — | — |
| Adult | -0.04 [-2.13, 2.05]_2_ | 0.01 | 13.64 |  | — | — | — |  | | — | | — | — |
| **Sex** |  |  |  |  |  |  |  |  | |  | |  |  |
| Female | -0.07 [-0.39, 0.25]_5_ | 0.00 | 3.83 |  | 0.09 [-0.14, 0.31]_4_ | 0.00 | 0.00 |  | | 0.10 [-0.08, 0.27]_3_ | | 0.00 | 0.00 |
| Male | 0.12 [-0.02, 0.25]_5_ | 0.00 | 0.00 |  | 0.13 [-0.39, 0.64]_4_ | 0.06 | 57.64 |  | | 0.03 [-0.42, 0.48]_3_ | | 0.00 | 0.00 |
| **Employment** |  |  |  |  |  |  |  |  | |  | |  |  |
| Unemployed | 0.06 [-0.70, 0.82]_2_ | 0.00 | 0.00 |  | — | — | — |  | | 0.11 [-1.89, 2.11]_2_ | | 0.01 | 24.33 |
| Employed | -0.01 [-0.75, 0.74]_2_ | 0.00 | 0.00 |  | — | — | — |  | | 0.04 [-0.11, 0.20]_2_ | | 0.00 | 0.00 |
| **Relationship Status** |  |  |  |  |  |  |  |  | |  | |  |  |
| Single | 0.09 [-0.11, 0.28]_3_ | 0.00 | 0.00 |  | -0.02 [-1.23, 1.19]_2_ | 0.00 | 0.00 |  | | 0.01 [-1.64, 1.67]_2_ | | 0.00 | 0.00 |
| Married | 0.17 [-0.17, 0.50]_3_ | 0.00 | 0.00 |  | 0.09 [-1.64, 1.83]_2_ | 0.00 | 0.00 |  | | 0.30 [-0.52, 1.11]_2_ | | 0.00 | 0.00 |
| **Education** |  |  |  |  |  |  |  |  | |  | |  |  |
| Below High School | 0.08 [-0.14, 0.30]_4_ | 0.00 | 0.00 |  | 0.11 [-0.14, 0.36]_3_ | 0.00 | 0.00 |  | | 0.15 [-0.90, 1.20]_2_ | | 0.00 | 0.00 |
| High School  Diploma or above | 0.12 [-0.15, 0.38]_3_ | 0.00 | 0.00 |  | -0.09 [-0.50, 0.32]_2_ | 0.00 | 0.00 |  | | -0.01 [-1.24, 1.22]_2_ | | 0.00 | 0.00 |
| *Note*. $\bar{ES}$ = average effect size (marginal mean difference between intervention and comparison conditions within a specific moderator subgroup). CI = 95% confidence interval with robust standard errors. — indicates results not available due to inadequate effect sizes. Subscripts indicate *k* (number of studies). | | | | | | | | | | | | | |

| **Supplemental Table 8** | | | | | | | | | | | | | |
| --- | --- | --- | --- | --- | --- | --- | --- | --- | --- | --- | --- | --- | --- |
| Pooled pretest-adjusted standardized effect sizes, 95% confidence intervals, and heterogeneity statistics by each moderator subgroup, for alcohol-related consequences at 3, 6, and 9-12-months post-baseline | | | | | | | | | | | | | |
|  | Follow-up Time | | | | | | | | | | | | |
|  | 3 month | | |  | 6 month | | | |  | | 9-12 months | | |
|  | $\bar{ES}$ [95% CI] | $\tau^{2}$ | $I^{2}$ (%) |  | $\bar{ES}$ [95% CI] | $\tau^{2}$ | $I^{2}$ (%) |  | | $\bar{ES}$ [95% CI] | | $\tau^{2}$ | $I^{2}$ (%) |
| **Age** |  |  |  |  |  |  |  |  | |  | |  |  |
| Adolescent | 0.06 [-0.31, 0.43]_2_ | 0.00 | 0.00 |  | — | — | — |  | | — | | — | — |
| Young adult | 0.11 [-0.27, 0.49]_5_ | 0.01 | 9.21 |  | 0.23 [-3.94, 4.39]_2_ | 0.14 | 63.80 |  | | 0.06 [-0.42, 0.54]_4_ | | 0.04 | 41.08 |
| Adult | 0.19 [-0.32, 0.69]_3_ | 0.00 | 0.00 |  | — | — | — |  | | 0.04 [-0.44, 0.53]_3_ | | 0.01 | 30.18 |
| **Sex** |  |  |  |  |  |  |  |  | |  | |  |  |
| Female | **0.16 [0.08, 0.25]_7,*_** | 0.00 | 0.00 |  | 0.08 [-0.14, 0.30]_5_ | 0.00 | 0.02 |  | | **0.15 [0.01, 0.29]_5_** | | 0.00 | 0.00 |
| Male | 0.09 [-0.01, 0.20]_7_ | 0.00 | 0.00 |  | 0.08 [-0.15, 0.31]_5_ | 0.01 | 25.92 |  | | 0.02 [-0.10, 0.13]_5_ | | 0.00 | 0.00 |
| **Education** |  |  |  |  |  |  |  |  | |  | |  |  |
| Below High School | 0.13 [-0.24, 0.49]_2_ | 0.00 | 0.00 |  | -0.04 [-0.41, 0.33]_3_ | 0.00 | 0.00 |  | | — | | — | — |
| High School  Diploma or above | 0.63 [-7.00, 8.25]_2_ | 0.58 | 80.15 |  | 0.55 [-0.64, 1.74]_3_ | 0.17 | 68.93 |  | | — | | — | — |
| *Note*. Results in boldface are significantly different from null value (i.e., indicating no marginal mean difference between intervention and comparison conditions within a specific moderator subgroup). * subscript indicates significant values after Benjamini-Hochberg adjustment for multiple comparisons. $\bar{ES}$ = average effect size. CI = 95% confidence interval with robust standard errors. — indicates results not available due to inadequate effect sizes. Subscripts indicate *k* (number of studies). | | | | | | | | | | | | | |

| **Supplemental Table 9** | | | | | | | | | | | | | |
| --- | --- | --- | --- | --- | --- | --- | --- | --- | --- | --- | --- | --- | --- |
| Pooled pretest-adjusted standardized effect sizes, 95% confidence intervals, and heterogeneity statistics by each moderator subgroup, for other drug-related consequences at 3, 6, and 9-12-months post-baseline | | | | | | | | | | | | | |
|  | Follow-up Time | | | | | | | | | | | | |
|  | 3 month | | |  | 6 month | | | |  | | 9-12 months | | |
|  | $\bar{ES}$ [95% CI] | $\tau^{2}$ | $I^{2}$ (%) |  | $\bar{ES}$ [95% CI] | $\tau^{2}$ | $I^{2}$ (%) |  | | $\bar{ES}$ [95% CI] | | $\tau^{2}$ | $I^{2}$ (%) |
| **Age** |  |  |  |  |  |  |  |  | |  | |  |  |
| Adolescent | 0.10 [-3.74, 3.94]_2_ | 0.12 | 57.36 |  | — | — | — |  | | — | | — | — |
| Young adult | **0.27 [0.12, 0.42]_2_** | 0.00 | 0.00 |  | 0.23 [-2.61, 3.07]_2_ | 0.03 | 28.12 |  | | — | | — | — |
| Adult | — | — | — |  | — | — | — |  | | — | | — | — |
| **Sex** |  |  |  |  |  |  |  |  | |  | |  |  |
| Female | 0.17 [-3.78, 4.11]_2_ | 0.11 | 47.07 |  | 0.06 [-0.05, 0.16]_2_ | 0.00 | 0.00 |  | | — | | — | — |
| Male | 0.01 [-0.80, 0.81]_2_ | 0.00 | 0.00 |  | 0.19 [-0.80, 1.18]_2_ | 0.00 | 0.00 |  | | — | | — | — |
| *Note*. Results in boldface are significantly different from null value (i.e., indicating no marginal mean difference between intervention and comparison conditions within a specific moderator subgroup). $\bar{ES}$ = average effect size. CI = 95% confidence interval with robust standard errors. — indicates results not available due to inadequate effect sizes. Subscripts indicate *k* (number of studies). | | | | | | | | | | | | | |

| **Supplemental Table 10** | | | | | | | | | | | | | |
| --- | --- | --- | --- | --- | --- | --- | --- | --- | --- | --- | --- | --- | --- |
| Pooled pretest-adjusted standardized effect sizes, 95% confidence intervals, and heterogeneity statistics by each moderator subgroup, for mental health symptoms at 3, 6, and 9-12-months post-baseline | | | | | | | | | | | | | |
|  | Follow-up Time | | | | | | | | | | | | |
|  | 3 month | | |  | 6 month | | | |  | | 9-12 months | | |
|  | $\bar{ES}$ [95% CI] | $\tau^{2}$ | $I^{2}$ (%) |  | $\bar{ES}$ [95% CI] | $\tau^{2}$ | $I^{2}$ (%) |  | | $\bar{ES}$ [95% CI] | | $\tau^{2}$ | $I^{2}$ (%) |
| **Age** |  |  |  |  |  |  |  |  | |  | |  |  |
| Adolescent | — | — | — |  | — | — | — |  | | — | | — | — |
| Young adult | -0.04 [-0.30, 0.23]_4_ | 0.00 | 0.00 |  | — | — | — |  | | 0.77 [-0.54, 2.08]_2_ | | 0.00 | 0.00 |
| Adult | 0.04 [-0.08, 0.16]_4_ | 0.00 | 0.00 |  | — | — | — |  | | 0.30 [-2.88, 3.49]_2_ | | 0.11 | 88.50 |
| **Sex** |  |  |  |  |  |  |  |  | |  | |  |  |
| Female | 0.23 [0.00, 0.46]_4_ | 0.00 | 0.00 |  | -0.23 [-1.98, 1.52]_2_ | 0.00 | 7.27 |  | | 0.50 [-1.11, 2.12]_2_ | | 0.01 | 12.15 |
| Male | -0.08 [-0.42, 0.26]_4_ | 0.01 | 25.20 |  | -0.02 [-2.75, 2.72]_2_ | 0.05 | 39.70 |  | | 0.37 [-4.80, 5.54]_2_ | | 0.30 | 91.08 |
| **Employment** |  |  |  |  |  |  |  |  | |  | |  |  |
| Unemployed | 0.03 [-0.02, 0.08]_4_ | 0.00 | 0.00 |  | — | — | — |  | | 0.38 [-3.25, 4.00]_2_ | | 0.13 | 80.42 |
| Employed | 0.03 [-0.25, 0.30]_4_ | 0.00 | 0.00 |  | — | — | — |  | | 0.32 [-3.28, 3.92]_2_ | | 0.13 | 82.05 |
| **Relationship Status** |  |  |  |  |  |  |  |  | |  | |  |  |
| Single | 0.01 [-0.04, 0.06]_2_ | 0.00 | 0.00 |  | — | — | — |  | | — | | — | — |
| Married | 0.19 [-5.01, 5.40]_2_ | 0.09 | 19.51 |  | — | — | — |  | | — | | — | — |
| **Education** |  |  |  |  |  |  |  |  | |  | |  |  |
| Below High School | -0.03 [-0.35, 0.28]_2_ | 0.00 | 0.00 |  | — | — | — |  | | — | | — | — |
| High School  Diploma or above | 0.01 [-0.84, 0.86]_2_ | 0.00 | 0.00 |  | — | — | — |  | | — | | — | — |
| **BL Severity Substance Use** |  |  |  |  |  |  |  |  | |  | |  |  |
| Low severity | -0.01 [-0.94, 0.92]_2_ | 0.00 | 0.00 |  | — | — | — |  | | — | | — | — |
| Moderate severity | 0.04 [-0.18, 0.26]_2_ | 0.00 | 0.00 |  | — | — | — |  | | — | | — | — |
| High severity | -0.24 [-1.67, 1.20]_2_ | 0.00 | 0.00 |  | — | — | — |  | | — | | — | — |
| *Note*. $\bar{ES}$ = average effect size (marginal mean difference between intervention and comparison conditions within a specific moderator subgroup). CI = 95% confidence interval with robust standard errors. — indicates results not available due to inadequate effect sizes. Subscripts indicate *k* (number of studies). | | | | | | | | | | | | | |

| **Supplemental Table 11** | | | | | | | | | | | | | |
| --- | --- | --- | --- | --- | --- | --- | --- | --- | --- | --- | --- | --- | --- |
| Pooled pretest-adjusted standardized effect sizes, 95% confidence intervals, and heterogeneity statistics by each moderator subgroup, for physical health symptoms at 3, 6, and 9-12-months post-baseline | | | | | | | | | | | | | |
|  | Follow-up Time | | | | | | | | | | | | |
|  | 3 month | | |  | 6 month | | | |  | | 9-12 months | | |
|  | $\bar{ES}$ [95% CI] | $\tau^{2}$ | $I^{2}$ (%) |  | $\bar{ES}$ [95% CI] | $\tau^{2}$ | $I^{2}$ (%) |  | | $\bar{ES}$ [95% CI] | | $\tau^{2}$ | $I^{2}$ (%) |
| **Age** |  |  |  |  |  |  |  |  | |  | |  |  |
| Adolescent | — | — | — |  | — | — | — |  | | — | | — | — |
| Young adult | -0.05 [-0.40, 0.30]^3^ | 0.00 | 0.00 |  | — | — | — |  | | 0.14 [-1.04, 1.32]^2^ | | 0.00 | 0.00 |
| Adult | 0.01 [-0.28, 0.29]^3^ | 0.00 | 0.00 |  | — | — | — |  | | 0.17 [-2.55, 2.89]^2^ | | 0.08 | 85.88 |
| **Sex** |  |  |  |  |  |  |  |  | |  | |  |  |
| Female | **0.11 [0.01, 0.20]_4_** | 0.00 | 0.00 |  | 0.09 [-0.26, 0.43]_3_ | 0.00 | 0.00 |  | | 0.19 [-0.20, 0.58]_3_ | | 0.01 | 35.32 |
| Male | -0.03 [-0.14, 0.09]_4_ | 0.00 | 0.00 |  | 0.06 [-0.39, 0.51]_3_ | 0.01 | 36.68 |  | | 0.03 [-0.38, 0.43]_3_ | | 0.00 | 10.99 |
| **Employment** |  |  |  |  |  |  |  |  | |  | |  |  |
| Unemployed | 0.02 [-0.22, 0.25]_3_ | 0.00 | 0.00 |  | — | — | — |  | | 0.16 [-2.76, 3.08]_2_ | | 0.07 | 70.68 |
| Employed | -0.07 [-0.37, 0.24]_3_ | 0.00 | 0.00 |  | — | — | — |  | | 0.15 [-1.94, 2.23]_2_ | | 0.03 | 44.19 |
| **Relationship Status** |  |  |  |  |  |  |  |  | |  | |  |  |
| Single | 0.05 [-0.19, 0.30]_3_ | 0.00 | 0.00 |  | 0.00 [-0.47, 0.46]_2_ | 0.00 | 0.00 |  | | -0.03 [-0.53, 0.47]_2_ | | 0.00 | 0.00 |
| Married | -0.21 [-0.67, 0.24]_3_ | 0.00 | 0.00 |  | -0.05 [-0.67, 0.57]_2_ | 0.00 | 0.00 |  | | 0.01 [-0.17, 0.19]_2_ | | 0.00 | 0.00 |
| **Education** |  |  |  |  |  |  |  |  | |  | |  |  |
| Below High School | -0.02 [-0.11, 0.06]_3_ | 0.00 | 0.00 |  | 0.00 [-0.97, 0.97]_2_ | 0.00 | 0.00 |  | | -0.11 [-0.27, 0.05]_2_ | | 0.00 | 0.00 |
| High School  Diploma or above | 0.03 [-0.16, 0.23]_3_ | 0.00 | 0.00 |  | -0.02 [-0.28, 0.25]_2_ | 0.00 | 0.00 |  | | 0.02 [-0.58, 0.63]_2_ | | 0.00 | 0.00 |
| **BL Severity Substance Use** | |  |  |  |  |  |  |  | |  | |  |  |
| Low severity | -0.07 [-1.57, 1.44]_2_ | 0.00 | 0.00 |  | — | — | — |  | | — | | — | — |
| Moderate severity | 0.03 [-2.11, 2.18]_2_ | 0.00 | 0.00 |  | — | — | — |  | | — | | — | — |
| High severity | -0.12 [-1.54, 1.30]_2_ | 0.00 | 0.00 |  | — | — | — |  | | — | | — | — |
| *Note*. Results in boldface are significantly different from null value (i.e., indicating no marginal mean difference between intervention and comparison conditions within a specific moderator subgroup). * subscript indicates significant values after Benjamini-Hochberg adjustment for multiple comparisons. $\bar{ES}$ = average effect size. CI = 95% confidence interval with robust standard errors. — indicates results not available due to inadequate effect sizes. Subscripts indicate *k* (number of studies). | | | | | | | | | | | | | |

| **Supplemental Table 12** | | | | | | | | | | | | | |
| --- | --- | --- | --- | --- | --- | --- | --- | --- | --- | --- | --- | --- | --- |
| Pooled pretest-adjusted standardized effect sizes, 95% confidence intervals, and heterogeneity statistics by each moderator subgroup, for substance use treatment utilization at 3, 6, and 9-12-months post-baseline | | | | | | | | | | | | | |
|  | Follow-up Time | | | | | | | | | | | | |
|  | 3 month | | |  | 6 month | | | |  | | 9-12 months | | |
|  | $\bar{ES}$ [95% CI] | $\tau^{2}$ | $I^{2}$ (%) |  | $\bar{ES}$ [95% CI] | $\tau^{2}$ | $I^{2}$ (%) |  | | $\bar{ES}$ [95% CI] | | $\tau^{2}$ | $I^{2}$ (%) |
| **Age** |  |  |  |  |  |  |  |  | |  | |  |  |
| Adolescent | — | — | — |  | — | — | — |  | | — | | — | — |
| Young adult | 0.34 [-0.72, 1.40]_2_ | 0.00 | 0.00 |  | 0.06 [-1.52, 1.64]_2_ | 0.00 | 0.00 |  | | -0.08 [-0.23, 0.08]_3_ | | 0.00 | 0.00 |
| Adult | 0.11 [-2.21, 2.44]_2_ | 0.05 | 68.64 |  | -0.01 [-1.09, 1.07]_2_ | 0.00 | 0.09 |  | | -0.12 [-0.36, 0.11]_3_ | | 0.00 | 0.00 |
| **Sex** |  |  |  |  |  |  |  |  | |  | |  |  |
| Female | **0.25 [0.21, 0.30]_3, *_** | 0.00 | 0.00 |  | 0.06 [-0.15, 0.26]_3_ | 0.00 | 0.00 |  | | 0.00 [-0.09, 0.09]_4_ | | 0.00 | 0.00 |
| Male | 0.06 [-0.44, 0.56]_3_ | 0.01 | 46.56 |  | -0.10 [-0.33, 0.14]_3_ | 0.00 | 0.00 |  | | -0.06 [-0.30, 0.18]_4_ | | 0.01 | 46.70 |
| **Relationship Status** |  |  |  |  |  |  |  |  | |  | |  |  |
| Single | 0.09 [-0.24, 0.42]_2_ | 0.00 | 0.00 |  | -0.08 [-0.32, 0.17]_3_ | 0.00 | 8.76 |  | | 0.01 [-0.22, 0.24]_3_ | | 0.00 | 0.00 |
| Married | -0.08 [-4.06, 3.89]_2_ | 0.13 | 61.20 |  | 0.02 [-0.53, 0.58]_3_ | 0.01 | 13.78 |  | | 0.00 [-0.42, 0.42]_3_ | | 0.00 | 0.00 |
| **Education** |  |  |  |  |  |  |  |  | |  | |  |  |
| Below High School | 0.11 [-0.52, 0.73]_2_ | 0.00 | 0.00 |  | -0.01 [-0.18, 0.16]_3_ | 0.00 | 0.00 |  | | -0.03 [-0.18, 0.12]_3_ | | 0.00 | 0.00 |
| High School  Diploma or above | 0.07 [-0.68, 0.83]_2_ | 0.00 | 0.00 |  | -0.12 [-0.29, 0.05]_2_ | 0.00 | 0.00 |  | | 0.00 [-0.36, 0.36]_3_ | | 0.01 | 40.21 |
| *Note*. Results in boldface are significantly different from null value (i.e., indicating no marginal mean difference between intervention and comparison conditions within a specific moderator subgroup). * subscript indicates significant values after Benjamini-Hochberg adjustment for multiple comparisons. $\bar{ES}$ = average effect size. CI = 95% confidence interval with robust standard errors. — indicates results not available due to inadequate effect sizes. Subscripts indicate *k* (number of studies). | | | | | | | | | | | | | |

| **Supplemental Table 13** | | | | | | | | | | | | | |
| --- | --- | --- | --- | --- | --- | --- | --- | --- | --- | --- | --- | --- | --- |
| Pooled pretest-adjusted standardized effect sizes, 95% confidence intervals, and heterogeneity statistics by each moderator subgroup, for emergency department utilization at 3, 6, and 9-12-months post-baseline | | | | | | | | | | | | | |
|  | Follow-up Time | | | | | | | | | | | | |
|  | 3 month | | |  | 6 month | | | |  | | 9-12 months | | |
|  | $\bar{ES}$ [95% CI] | $\tau^{2}$ | $I^{2}$ (%) |  | $\bar{ES}$ [95% CI] | $\tau^{2}$ | $I^{2}$ (%) |  | | $\bar{ES}$ [95% CI] | | $\tau^{2}$ | $I^{2}$ (%) |
| **Age** |  |  |  |  |  |  |  |  | |  | |  |  |
| Adolescent | — | — | — |  | — | — | — |  | | — | | — | — |
| Young adult | -0.12 [-0.97, 0.73]_2_ | 0.00 | 0.00 |  | — | — | — |  | | — | | — | — |
| Adult | -0.06 [-0.80, 0.68]_2_ | 0.00 | 0.00 |  | — | — | — |  | | — | | — | — |
| **Sex** |  |  |  |  |  |  |  |  | |  | |  |  |
| Female | -0.02 [-0.26, 0.23]_3_ | 0.00 | 0.00 |  | **-0.13 [-0.20, -0.06]_2_** | 0.00 | 0.00 |  | | 0.05 [-1.20, 1.30]_2_ | | 0.00 | 0.00 |
| Male | -0.04 [-0.13, 0.05]_3_ | 0.00 | 0.00 |  | 0.00 [-0.34, 0.34]_2_ | 0.00 | 0.00 |  | | -0.09 [-0.80, 0.62]_2_ | | 0.00 | 0.00 |
| **Employment** |  |  |  |  |  |  |  |  | |  | |  |  |
| Unemployed | -0.12 [-1.04, 0.80]_2_ | 0.00 | 0.00 |  | — | — | — |  | | — | | — | — |
| Employed | 0.04 [-0.42, 0.49]_2_ | 0.00 | 0.00 |  | — | — | — |  | | — | | — | — |
| **Relationship Status** |  |  |  |  |  |  |  |  | |  | |  |  |
| Single | 0.00 [-0.80, 0.80]_2_ | 0.00 | 0.00 |  | -0.04 [-0.08, 0.00]_2_ | 0.00 | 0.00 |  | | -0.09 [-0.71, 0.53]_2_ | | 0.00 | 0.00 |
| Married | -0.09 [-1.55, 1.37]_2_ | 0.00 | 0.00 |  | -0.02 [-0.88, 0.84]_2_ | 0.00 | 0.00 |  | | **0.25 [0.18, 0.31]_2_** | | 0.00 | 0.00 |
| **Education** |  |  |  |  |  |  |  |  | |  | |  |  |
| Below High School | -0.10 [-0.24, 0.04]_3_ | 0.00 | 0.00 |  | — | — | — |  | | -0.09 [-1.26, 1.09]_2_ | | 0.00 | 0.00 |
| High School  Diploma or above | -0.02 [-0.24, 0.20]_3_ | 0.00 | 0.00 |  | — | — | — |  | | -0.02 [-0.61, 0.58]_2_ | | 0.00 | 0.00 |
| *Note*. Results in boldface are significantly different from null value (i.e., indicating no marginal mean difference between intervention and comparison conditions within a specific moderator subgroup). $\bar{ES}$ = average effect size. CI = 95% confidence interval with robust standard errors. — indicates results not available due to inadequate effect sizes. Subscripts indicate *k* (number of studies). | | | | | | | | | | | | | |

| **Supplemental Table 14** | | | | | | | | | | | | | |
| --- | --- | --- | --- | --- | --- | --- | --- | --- | --- | --- | --- | --- | --- |
| Pooled pretest-adjusted standardized effect sizes, 95% confidence intervals, and heterogeneity statistics by each moderator subgroup, for readiness to change at 3, 6, and 9-12-months post-baseline | | | | | | | | | | | | | |
|  | Follow-up Time | | | | | | | | | | | | |
|  | 3 month | | |  | 6 month | | | |  | | 9-12 months | | |
|  | $\bar{ES}$ [95% CI] | $\tau^{2}$ | $I^{2}$ (%) |  | $\bar{ES}$ [95% CI] | $\tau^{2}$ | $I^{2}$ (%) |  | | $\bar{ES}$ [95% CI] | | $\tau^{2}$ | $I^{2}$ (%) |
| **Age** |  |  |  |  |  |  |  |  | |  | |  |  |
| Adolescent | — | — | — |  | — | — | — |  | | — | | — | — |
| Young adult | -0.11 [-0.35, 0.13]_6_ | 0.00 | 0.00 |  | — | — | — |  | | 0.01 [-0.01, 0.04]_3_ | | 0.00 | 0.00 |
| Adult | -0.02 [-0.26, 0.22]_5_ | 0.02 | 31.05 |  | — | — | — |  | | -0.05 [-0.39, 0.29]_3_ | | 0.00 | 5.60 |
| **Sex** |  |  |  |  |  |  |  |  | |  | |  |  |
| Female | 0.07 [-0.14, 0.27]_6_ | 0.00 | 0.00 |  | — | — | — |  | | 0.11 [-0.35, 0.58]_3_ | | 0.00 | 0.00 |
| Male | -0.07 [-0.27, 0.13]_6_ | 0.00 | 28.21 |  | — | — | — |  | | -0.07 [-0.35, 0.20]_3_ | | 0.00 | 0.00 |
| **Employment** |  |  |  |  |  |  |  |  | |  | |  |  |
| Unemployed | 0.04 [-0.26, 0.34]_4_ | 0.00 | 0.00 |  | — | — | — |  | | — | | — | — |
| Employed | **0.11 [0.01, 0.22]_4_** | 0.00 | 0.00 |  | — | — | — |  | | — | | — | — |
| **Relationship Status** |  |  |  |  |  |  |  |  | |  | |  |  |
| Single | 0.13 [-0.63, 0.89]_3_ | 0.00 | 0.00 |  | — | — | — |  | | — | | — | — |
| Married | -0.27 [-3.49, 2.94]_3_ | 0.70 | 56.91 |  | — | — | — |  | | — | | — | — |
| **Education** |  |  |  |  |  |  |  |  | |  | |  |  |
| Below High School | 0.07 [-0.25, 0.38]_4_ | 0.00 | 0.00 |  | — | — | — |  | | — | | — | — |
| High School  Diploma or above | 0.14 [-0.15, 0.43]_4_ | 0.00 | 0.00 |  | — | — | — |  | | — | | — | — |
| **BL Severity Substance Use** | |  |  |  |  |  |  |  | |  | |  |  |
| Low severity | 0.03 [-0.78, 0.84]_2_ | 0.00 | 0.00 |  | — | — | — |  | | — | | — | — |
| Moderate severity | 0.05 [-0.60, 0.70]_2_ | 0.00 | 0.00 |  | — | — | — |  | | — | | — | — |
| High severity | -0.12 [-0.89, 0.65]_2_ | 0.00 | 0.00 |  | — | — | — |  | | — | | — | — |
| *Note*. Results in boldface are significantly different from null value (i.e., indicating no marginal mean difference between intervention and comparison conditions within a specific moderator subgroup). $\bar{ES}$ = average effect size. CI = 95% confidence interval with robust standard errors. — indicates results not available due to inadequate effect sizes. Subscripts indicate *k* (number of studies). | | | | | | | | | | | | | |

| **Supplemental Table 15**  Descriptive comparison of characteristics of studies and interventions included in qualitative and quantitative aggregate data minus the individual participant data samples and individual participant data syntheses (AD-IPD *k* = 87; IPD *k* = 29). | | | | | | | |  |  |
| --- | --- | --- | --- | --- | --- | --- | --- | --- | --- |
| Study and design characteristics | % (*n*) ^a^ | | |  | | Intervention features | % (*n*) ^a^ | |  |
|  | AD-IPD | IPD | | | *p* ^g^ |  | AD-IPD | IPD | *p* ^g^ |
| Country/region ^b^ |  |  |  | | > .05 | Setting ^b^ |  |  | > .05 |
| Asia | 5 (4) | 3 (1) | | |  | Emergency department | 44 (38) | 34 (10) |  |
| Australia/New Zealand | 9 (8) | 3 (1) | | |  | Community | 32 (28) | 39 (11) |  |
| South Africa | 5 (4) | 3 (1) | | |  | University | 22 (19) | 21 (6) |  |
| South America | 1 (1) | 7 (2) | | |  | Outpatient | 10 (9) | 14 (4) |  |
| U.S./Canada | 69 (52) | 55 (16) | | |  | Inpatient | 5 (4) | 7 (2) |  |
| *U.S. Midwest* | 17 (15) | - |  | |  | Private provider | 10 (9) | 7 (2) |  |
| *U.S. Northeast* | 24 (21) | 28 (8) | | |  | Student health center | 8 (7) | 0 (0) |  |
| *U.S. South* | 6 (5) | 7 (2) | | |  | Other | 22 (19) | 7 (2) |  |
| *U.S. West* | 18 (10) | 19 (4) |  | |  | Modality ^c^ |  |  | > .05 |
| *Multiple U.S. regions* | 3 (3) | - |  | |  | In-person | 72 (63) | 86 (25) |  |
| Western Europe | 18 (16) | 28 (8) | | |  | Computer/tablet/smartphone | 25 (22) | 7 (2) |  |
| Multiple | 2 (2) | - |  | |  | Telephone | 5 (4) | 7 (2) |  |
| Sample type ^b^ |  |  |  | | > .05 | Booster ^c^ |  |  |  |
| Screened/elevated risk | 98 (85) | 93 (27) | | |  | Booster delivered | 38 (33) | 24 (7) | > .05 |
| Universal/unscreened | 2 (2) | 7 (2) | | |  | No. boosters; median (range) ^d^ | 1 (1–4) | 1 (1-2) |  |
| Design ^b^ |  |  |  | | > .05 | Duration (minutes); *M* (*SD*) ^c^ | 27.3 (26.2) | 21.0 (19.5) |  |
| RCT | 90 (78) | 90 (26) | | |  | Components ^c, e^ |  |  | > .05 |
| Cluster RCT | 10 (9) | 10 (3) |  | |  | Advice | 67 (58) | 59 (17) |  |
| Comparison group type ^c^ |  |  |  | | > .05 | Information booklet | 63 (55) | 55 (16) |  |
| Treatment as usual/usual care | 44 (38) | 59 (17) | | |  | Decisional balance exercise | 37 (27) | 41 (12) |  |
| General health booklet | 39 (34) | 24 (7) | | |  | Goal-setting exercise | 54 (47) | 66 (19) |  |
| Sham intervention | 6 (5) | 10 (3) | | |  | Homework activity | 6 (5) | 0 (0) |  |
| No pretest assessment usual care | 5 (4) | - | | |  | Personalized normative feedback | 78 (68) | 72 (21) |  |
| Other | 14 (12) | 7 (2) |  | |  | Training | 18 (16) | 10 (3) |  |
| **Table 1, cont.** | | | | | | | | |  |
| Attrition; *M* (*SD*) |  |  |  | |  | Referrals | 25 (22) | 28 (8) |  |
| Overall ^b^ | 0.23 (0.15) | 0.22 (0.12) | | |  | Video | 3 (3) | 7 (2) |  |
| Differential ^c^ | 0.06 (0.05) | 0.05 (0.04) | | |  | Website | 6 (5) | 3 (1) |  |
| Implementation monitoring |  |  |  | | = .07 | Other | 38 (33) | 45 (13) |  |
| Yes | 56 (49) | 76 (22) | | |  | Provider characteristics | % (*n*) ^a^ | | > .05 |
| No | 3 (3) | 7 (2) | | |  | Typical provider ^c^ |  |  |  |
| Not reported | 40 (35) | 17 (5) | | |  | General practitioner (non-primary provider) | 11 (10) | 7 (2) |  |
| Implementation problems |  |  |  | | > .05 | Primary care provider | 20 (17) | 21 (6) |  |
| Yes | 17 (15) | 3 (1) | | |  | Behavioral specialist | 26 (23) | 38 (11) |  |
| Possible | 17 (15) | 21 (6) | | |  | Other specialist provider | 2 (2) | 0 (0) |  |
| Not reported | 66 (57) | 76 (22) | | |  | Peer | 8 (7) | 7 (2) |  |
| Intention-to-treat analysis |  |  | | | = .08 | Graduate student/trainee | 1 (1) | 3 (1) |  |
| Yes | 47 (41) | 84 (18) | | |  | Other provider | 39 (34) | 24 (7) |  |
| Possible | 21 (18) | 28 (8) | | |  |  |  |  |  |
| No | 31 (27) | 10 (3) | | |  |  |  |  |  |
| CONSORT diagram |  |  | | | > .05 |  |  |  |  |
| Yes | 78 (68) | 90 (26) | | |  |  |  |  |  |
| No | 22 (19) | 10 (3) | | |  |  |  |  |  |
| Study and design characteristics | % (*n*) ^a^ | | | |  | Provider characteristics | % (*n*) ^a^ | |  |
| Overall risk of bias |  |  | | | < .05 | Provider profession ^c^ |  |  | > .05 |
| Unclear | 70 (61) | 90 (26) | | |  | Medical doctor | 23 (20) | 21 (6) |  |
| High | 30 (26) | 10 (3) | | |  | Physician’s assistant | 1 (1) | 0 (0) |  |
| Participant characteristics | *M* (*SD*) ^f^ | | | |  | Nurse | 14 (12) | 7 (2) |  |
| Average age ^c^ | 36.2 (12.8) | 32.6 (13.5) | | | > .05 | Other medical specialist | 1 (1) | 3 (1) |  |
| Sample age group, % (*n*) |  |  | | | > .05 | Psychologist | 6 (5) | 17 (5) |  |
| Adolescent/young adult | 22 (19) | 31.0 (9) | | |  | Social worker | 7 (6) | 7 (2) |  |
| Mixed or adult only | 78 (68) | 69.0 (20) | | |  | Other behavioral health specialist | 15 (13) | 45 (13) |  |
| Percent female composition ^c^ | 37.3 (22.1) | 42.4 (19.7) | | | > .05 |  |  |  |  |
| **Table 1, cont.** |  |  | | |  |  |  |  |  |
| Race/ethnicity composition ^c^ |  |  | | | < .05 |  |  |  |  |
| Percent Asian | 12.8 (29.3) | 18.3 (40.0) | | |  |  |  |  |  |
| Percent Black | 32.2 (26.1) | 41.3 (27.9) | | |  |  |  |  |  |
| Percent Latinx | 29.9 (26.2) | 38.2 (34.3) | | |  |  |  |  |  |
| Percent White | 53.8 (29.5) | 41.8 (31.4) | | |  |  |  |  |  |
| *Notes. k* = number of studies. AD = aggregate data. IPD = individual participant data. ^a^ Percentages and counts shown unless otherwise indicated. ^b^ Estimates calculated at study level. ^c^ Estimates calculated at intervention or comparison group level, as appropriate. ^d^ Number (No.) of boosters calculated only among studies delivering boosters; one study (Córdoba et al., 1998) provided a variable number of boosters and is not included in estimates. ^e^ Interventions could use multiple components; percentages reflect proportion of all intervention groups using each component. ^f^ Means and standard deviations shown unless otherwise indicated. ^g^ chi-square or *t­-*tests used to compare the AD-IPD (those AD studies not included in the IPD meta-analysis) and IPD sample. *p*-value reflects statistical significance of comparisons, adjusted for multiple comparisons within each outcome domain. | | | | | | | |  |  |

| **Supplemental Table 16**  *Participants Available for Each Candidate Moderator* | | | | | | | | | | | | | | | | |
| --- | --- | --- | --- | --- | --- | --- | --- | --- | --- | --- | --- | --- | --- | --- | --- | --- |
| Moderator Domain | Participants in Each Domain  % (*n*) | | Number of Studies Reporting Outcome  *k* (%)^a^ | | | | | | | | | | | | | |
|  | Intervention  (*n* = 6, 577) | Control  (*n* = 5,497) | Binge Alcohol Use | Frequency Alcohol Use | Quantity Alcohol Use | Frequency Cannabis Use | Quantity Cannabis Use | Tobacco Use | Other Drug Use | Alcohol Consequences | Other Drug Consequences | Mental Health Symptoms | Physical Health Symptoms | Substance Use Treatment Use | ED Use | Readiness to Change |
| Age^b^ |  |  |  |  |  |  |  |  |  |  |  |  |  |  |  |  |
| Adolescent | 6 (250) | 5 (237) | 3 (10) | 3 (10) | - | 3 (10) | 3 (10) | - | - | 2 (7) | 2 (7) | - |  | - | - | - |
| Young Adult | 27 (1076) | 26 (1099) | 8 (28) | 9 (31) | 10 (34) | 5 (17) | 4 (14) | 4 (14) | 3 (10) | 5 (17) | 2 (7) | 4 (14) | 3 (10) | 2 (7) | 2 (7) | 6 (21) |
| Adult | 67 (2696) | 69 (2949) | 5 (17) | 6 (21) | 9 (31) | 2 (7) | - | 3 (10) | 2 (7) | 3 (10) | - | 4 (14) | 3 (10) | 2 (7) | 2 (7) | 5 (17) |
| Sex |  |  |  |  |  |  |  |  |  |  |  |  |  |  |  |  |
| Female | 43 (2,440) | 45 (2,229) | 10 (34) | 11 (38) | 11 (38) | 6 (21) | 5 (17) | 6 (21) | 5 (17) | 7 (24) | 2 (7) | 4 (14) | 4 (14) | 4 (14) | 3 (10) | 6 (21) |
| Male | 57 (3,213) | 55 (2,690) | 10 (34) | 11 (38) | 11 (38) | 6 (21) | 5 (17) | 6 (21) | 5 (17) | 7 (24) | 2 (7) | 4 (14) | 4 (14) | 4 (14) | 3 (10) | 6 (21) |
| Employment |  |  |  |  |  |  |  |  |  |  |  |  |  |  |  |  |
| Employed | 43 (692) | 39 (354) | 3 (10) | 4 (14) | 5 (17) | - | - | - | 2 (7) | - | - | 4 (14) | 3 (10) | - | 2 (7) | 4 (14) |
| Unemployed | 57 (910) | 61 (551) | 3 (10) | 4 (14) | 5 (17) | - | - | - | 2 (7) | - | - | 4 (14) | 3 (10) | - | 2 (7) | 4 (14) |
| Housing |  |  |  |  |  |  |  |  |  |  |  |  |  |  |  |  |
| Stable housing | 81 (456) | 80 (293) | - | - | - | - | - | - | - | - | - | - | - | - | - | - |
| Unstable housing | 19 (109) | 20 (72) | - | - | - | - | - | - | - | - | - | - | - | - | - | - |
| Relationship Status^c^ |  |  |  |  |  |  |  |  |  |  |  |  |  |  |  |  |
| Single | 68 (2,672) | 66 (1,837) | 4 (14) | 4 (14) | 5 (17) | - | - | - | 3 (10) | - | - | 2 (7) | 3 (10) | 3 (10) | 3 (10) | 3 (10) |
| Married | 32 (1,284) | 34 (937) | 4 (14) | 4 (14) | 5 (17) | - | - | - | 3 (10) | - | - | 2 (7) | 3 (10) | 3 (10) | 3 (10) | 3 (10) |
| Education |  |  |  |  |  |  |  |  |  |  |  |  |  |  |  |  |
| HS education or above | 63 (2,149) | 61 (1,887) | 5 (17) | 6 (21) | 5 (17) | 2 (7) | - | 2 (7) | 4 (14) | 2 (7) | - | 2 (7) | 3 (10) | 3 (10) | 3 (10) | 4 (14) |
| Below HS education | 37 (1,298) | 39 (1,229) | 5 (17) | 6 (21) | 5 (17) | 2 (7) | - | 2 (7) | 3 (10) | 2 (7) | - | 2 (7) | 3 (10) | 3 (10) | 3 (10) | 4 (14) |
| Baseline severity^d^ |  |  |  |  |  |  |  |  |  |  |  |  |  |  |  |  |
| Low severity | 58 (1006) | 48 (371) | 3 (10) | 3 (10) | 3 (10) | 3 (10) | - | 3 (10) | - | - | - | 2 (7) | 2 (7) | - | - | 2 (7) |
| Moderate severity | 31 (533) | 38 (291) | 3 (10) | 3 (10) | 3 (10) | 3 (10) | - | 3 (10) | - | - | - | 2 (7) | 2 (7) | - | - | 2 (7) |
| High severity |  |  | 2 (7) | 2 (7) | 3 (10) | 3 (10) | - | 2 (7) | - | - | - | 2 (7) | 2 (7) | - | - | 2 (7) |

*Note.* Intervention and Control conditions include all groups for reach primary study (e.g., including two intervention or control groups where applicable). ED = Emergency Department; HS = High School.

^a^ Study counts indicate the number of studies reporting an outcome at one or more follow-up times.

^b^ Adolescent < 18; Young Adult = 18 – 25; Adult 26 or older.

^c^ Single includes single, widowed, divorced, or otherwise unpartnered; Married includes married or in a committed relationship.

^b^ Low BL Severity = AUDIT score 1-7 or ASSIST total score 1-3; Moderate BL Severity = AUDIT score 8-15 or ASSIST total score 4-26; High BL Severity AUDIT score 16 or above or ASSIST total score 27 or above.
