# Supplementary material for: Investigating for Whom Brief Substance Use Interventions Are Most Effective: An Individual Participant Data Meta-analysis
Source: Prev Sci. 2023 May 3;24(8):1459–82. doi: 10.1007/s11121-023-01525-1 (PMC10678844; doi:10.1007/s11121-023-01525-1)

**Publication Bias Results**

The Orwin fail-safe N test estimates the number of studies needed to reduce the aggregate effect size to a specified critical value (Orwin, 1983), which was defined as 50% of the aggregate effect size in this study. We used this test to assess publication bias for each meta-analysis included in this study that resulted in a statistically significant result. The results of these tests follow.

For binge alcohol consumption at 3-months post-treatment for females, Orwin’s modified fail-safe N indicated that 10 studies would be required to reduce the aggregate effect size by 50%. For the quantity of alcohol consumption at 3-months post-treatment for females, Orwin’s modified fail-safe N indicated that 11 studies would be required to reduce the aggregate effect size by 50%. For the frequency of alcohol consumption at 3-months post-treatment for females, Orwin’s modified fail-safe N indicated that 11 studies would be required to reduce the aggregate effect size by 50%. For alcohol-related consequences at 3-months post-treatment for females, Orwin’s modified fail-safe N indicated that 7 studies would be required to reduce the aggregate effect size by 50%. Last, for the frequency of alcohol consumption at 3-months post-treatment for participants with below a High School level of education, Orwin’s modified fail-safe N indicated that 6 studies would be required to reduce the aggregate effect size by 50%. Notably, the average effect size for all five of these outcomes was quite small, as was the *k* of studies included (*k* ranges from 6-11), thus, results of these tests should be interpreted with caution. Additionally, the Orwin’s fail-safe N test assumes that the average effect of studies that are not included is null.

The following pages include funnel plots assessing for publication bias for each of the five main meta-analyses and findings in this paper.

**Binge Alcohol Consumption at 3-months Post-Treatment for Females**


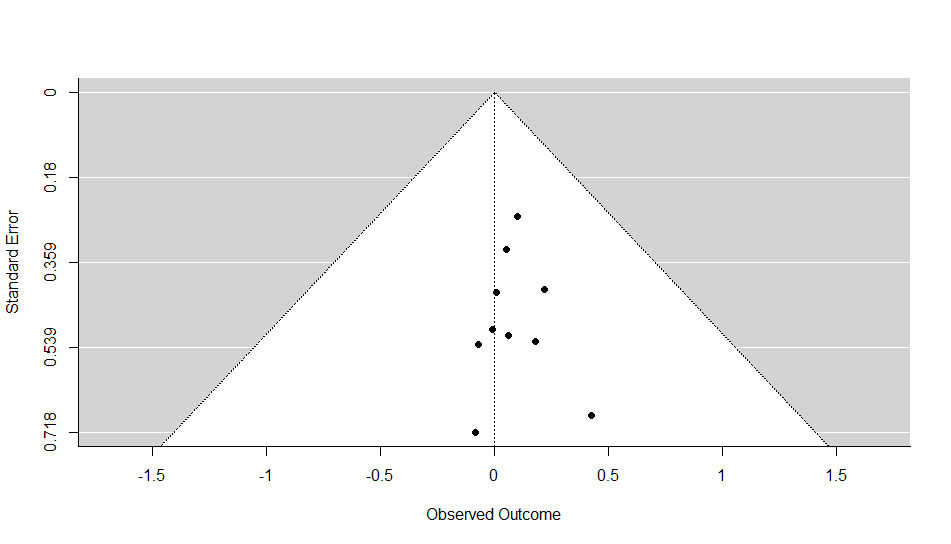


**Quantity of Alcohol Consumption at 3-months Post-Treatment for Females**


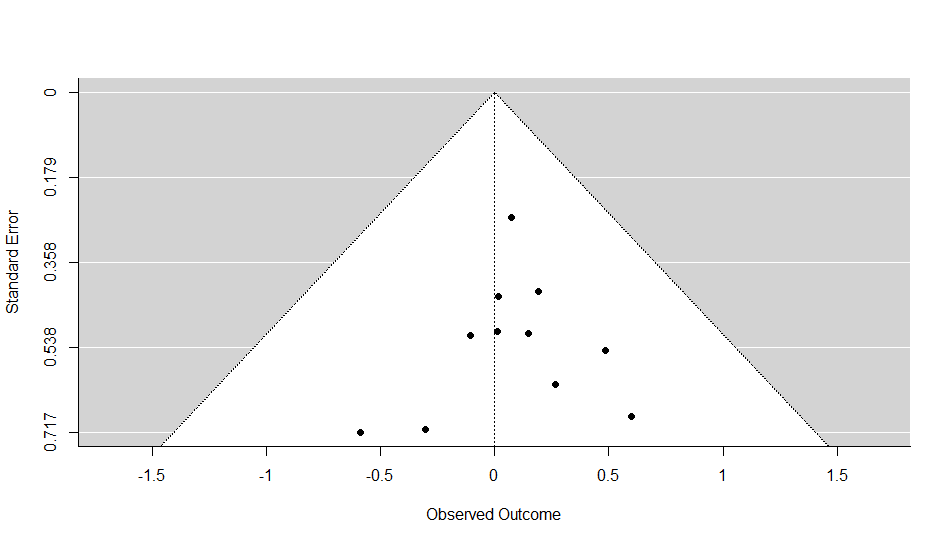


**Frequency of Alcohol Consumption at 3-months Post-Treatment for Females**


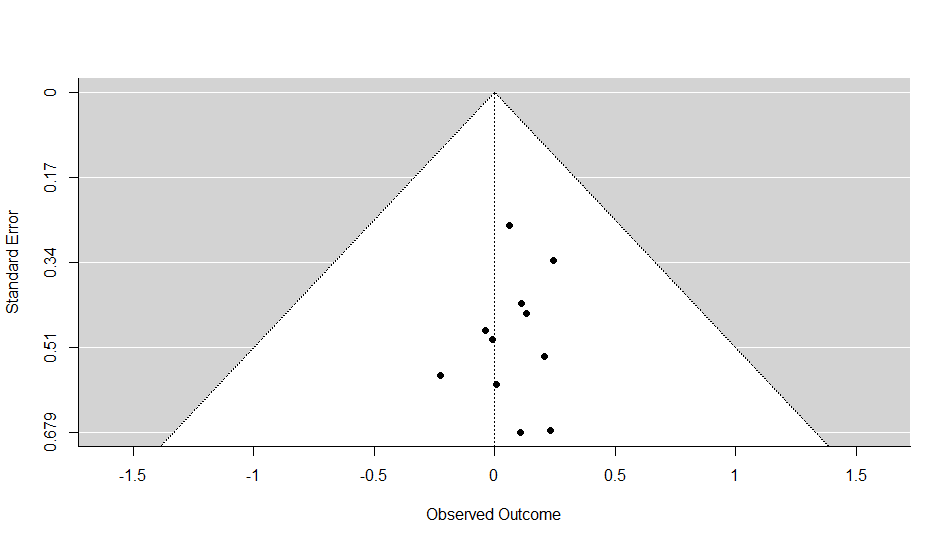


**Alcohol-related Consequences at 3-months Post-Treatment for Females**

**
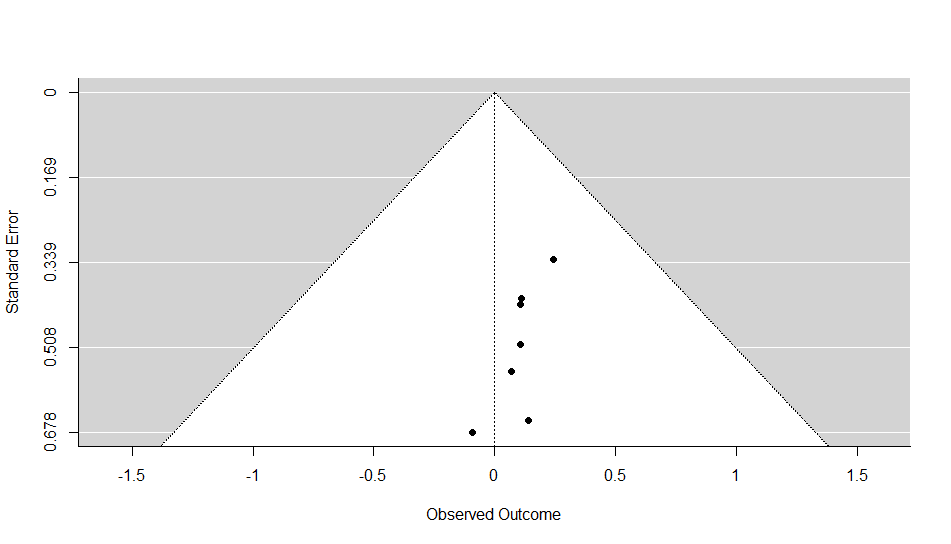
**

**Alcohol Consumption at 3-months Post-Treatment for Participants with Below a High School Level of Education**


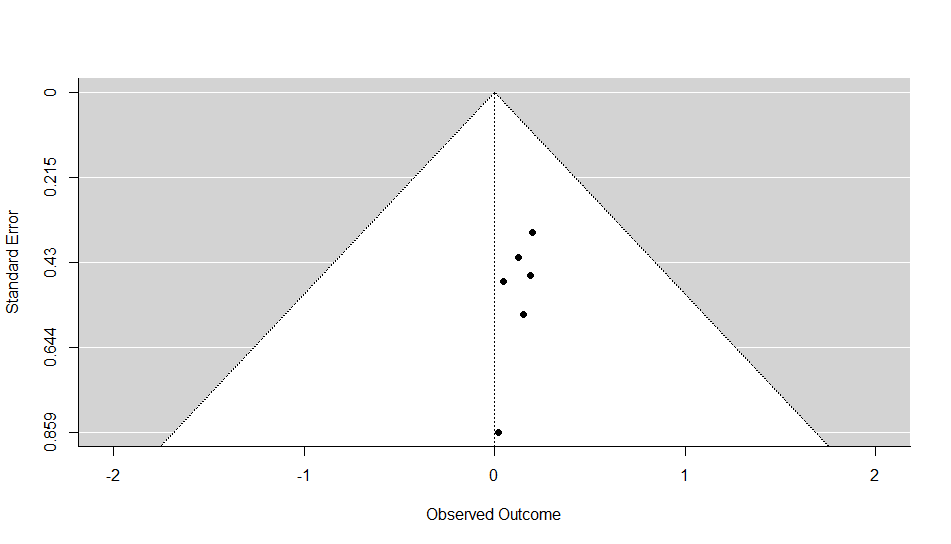

Supplement: Supplementary file 8 — Supplementary file8 (DOCX 87 KB) [file 11121_2023_1525_MOESM8_ESM.docx]
